# ATPase Subdomain IA Is a Mediator of Interdomain Allostery in Hsp70 Molecular Chaperones

**DOI:** 10.1371/journal.pcbi.1003624

**Published:** 2014-05-15

**Authors:** Ignacio J. General, Ying Liu, Mandy E. Blackburn, Wenzhi Mao, Lila M. Gierasch, Ivet Bahar

**Affiliations:** 1 Department of Computational and Systems Biology, School of Medicine, University of Pittsburgh, Pittsburgh, Pennsylvania, United States of America; 2 Department of Biochemistry & Molecular Biology, University of Massachusetts Amherst, Amherst, Massachusetts, United States of America; 3 Department of Pharmacology, Tsinghua University, Beijing, China; 4 Department of Chemistry, University of Massachusetts Amherst, Amherst, Massachusetts, United States of America; Chapman University, United States of America

## Abstract

The versatile functions of the heat shock protein 70 (Hsp70) family of molecular chaperones rely on allosteric interactions between their nucleotide-binding and substrate-binding domains, NBD and SBD. Understanding the mechanism of interdomain allostery is essential to rational design of Hsp70 modulators. Yet, despite significant progress in recent years, how the two Hsp70 domains regulate each other's activity remains elusive. Covariance data from experiments and computations emerged in recent years as valuable sources of information towards gaining insights into the molecular events that mediate allostery. In the present study, conservation and covariance properties derived from both sequence and structural dynamics data are integrated with results from Perturbation Response Scanning and *in vivo* functional assays, so as to establish the dynamical basis of interdomain signal transduction in Hsp70s. Our study highlights the critical roles of SBD residues D481 and T417 in mediating the coupled motions of the two domains, as well as that of G506 in enabling the movements of the α-helical lid with respect to the β-sandwich. It also draws attention to the distinctive role of the NBD subdomains: Subdomain IA acts as a key mediator of signal transduction between the ATP- and substrate-binding sites, this function being achieved by a cascade of interactions predominantly involving conserved residues such as V139, D148, R167 and K155. Subdomain IIA, on the other hand, is distinguished by strong coevolutionary signals (with the SBD) exhibited by a series of residues (D211, E217, L219, T383) implicated in DnaJ recognition. The occurrence of coevolving residues at the DnaJ recognition region parallels the behavior recently observed at the nucleotide-exchange-factor recognition region of subdomain IIB. These findings suggest that Hsp70 tends to adapt to co-chaperone recognition and activity via coevolving residues, whereas interdomain allostery, critical to chaperoning, is robustly enabled by conserved interactions.

## Author Summary

The Hsp70 family of molecular chaperones assists in protein folding, degradation, assembly/disassembly of some complexes, and intracellular trafficking. These activities in the cell are accomplished by coupled conformational switches/signals between their nucleotide-binding and substrate-binding domains (NBD and SBD), assisted by cognate co-chaperones. Despite significant progress in the field, the molecular basis of Hsp70 machinery and the key interactions that regulate interdomain communication are not fully understood. Using a combination of experimental and computational methods, including *in vivo* functional assays, sequence- and structure-based analyses and perturbation response scanning, we identified a network of conserved interactions in subdomain IA of the NBD, which plays a key (*effector*) role in propagating signals between the ATP-binding and substrate-binding sites. Subdomain IIA, on the other hand, appears to adapt to J-domain co-chaperone binding by virtue of a broadly distributed cluster of co-evolving residues on the recognition surface.

## Introduction

The heat shock protein 70 (Hsp70) family of molecular chaperones plays a key role in the quality control of protein folding, as well as in regulation of intracellular trafficking [1]–[3]. Hsp70 dysfunction has been implicated in a broad range of conditions/disorders including tumor growth and Alzheimer's disease [4], [5].

**Figure 1:**
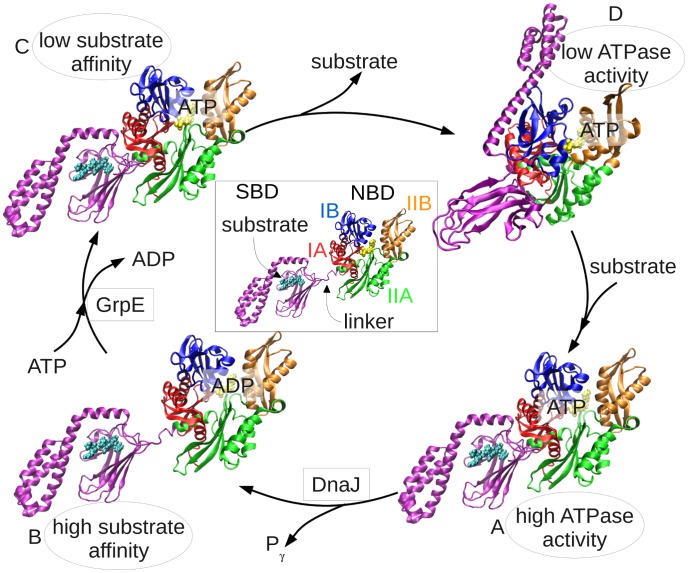
Hsp70 allosteric cycle. In the ADP-bound state (**B**, *bottom-left*), the SBD (*purple*) and NBD are loosely connected by a flexible interdomain linker. NBD subdomains are colored *red* (subdomain IA; residues 3-38; 112-184), *blue* (IB; residues 39-111), *green* (IIA; residues 185-228; 310-388) and *orange* (IIB; residues 229-309) as indicated in the middle diagram. Upon replacement of ADP by ATP (both in *yellow*, *space-filling*) and ensuing release of substrate (*cyan, space-filling*), an open-SBD conformer is assumed (**D**, *upper-right)*, where the α-helical lid is docked onto the NBD, exposing and opening the substrate-binding site. The two domains allosterically regulate each other through intermediate allosterically active states illustrated in **A** and **C** [8]: ATP binding decreases the substrate-binding affinity of the SBD; substrate-binding increases the ATPase activity of the NBD. The co-chaperones, DnaJ and GrpE, assist in the hydrolysis and nucleotide exchange steps, respectively. Diagrams **B** and **D** were generated using the PDB files 1DKX [7] and 4B9Q [25], respectively. **A** and **C** were generated manually—as their complete structure is unknown—based on the two mentioned structures and also on 1DKG [65] and 2KHO [16].

The versatile functions of Hsp70s rely on the allosteric interaction of their two domains, the ATPase domain, also referred to as the nucleotide-binding domain, NBD [6], and the substrate-binding domain, SBD [7]. **Figure 1** provides a schematic description of the *E. coli* Hsp70, DnaK, allosteric cycle that underlies its chaperoning activity [8], [9]: substrate binding (step **D**→**A**) promotes ATP hydrolysis at the NBD (stimulated by the co-chaperone DnaJ; **A**→**B**), which, in turn, induces a major conformational change in the SBD to stabilize the substrate and thus reduce its release and exchange rate/probability. The ADP produced upon ATP hydrolysis is then released and a new ATP molecule binds (**B**→**C** passage); nucleotide exchange is assisted by a nucleotide exchange factor (NEF, co-chaperone GrpE) [10]. This process enhances the release of substrate (**C**→**D**) [3]. Substrate release involves a large structural change in the SBD converting the chaperone back into its low ATPase activity state (conformer **D**). In the present work, we have explored properties of conformer **D**, in an effort to deduce the origins of interdomain communication.

Understanding the molecular events that control allosteric interactions is a challenge in general [11]–[14], and the allosteric mechanism of Hsp70's molecular machinery remains to be established, despite considerable progress made in recent years [8], [15]–[26]. The goal of the present study is to provide new insights into the mechanism of Hsp70 allostery, building on recent work in one of our laboratories [8], [17], [19], new structural data [25], [26], and recently developed computational approaches [27]. We use a combination of experimental and computational studies including *in vivo* functional assays, fluorescence assays, perturbation-response scanning (PRS) [28], information theoretic analysis for detecting conservation and coevolution patterns [29], the Gaussian Network Model (GNM) [30], [31] for characterizing collective movements and associated hinge sites, and identifying conserved or correlated residues that act as *sensors* or *effectors* of allosteric signals elicited by co-chaperone, nucleotide or substrate binding.

Our study reveals that NBD subdomain IA, and in particular a number of highly conserved (V139, D148, K155, R167, N170, E171) or co-evolving (R159, L177) residues therein, serve as mediators of communication between the substrate- and nucleotide-binding sites of the respective SBD and NBD, in addition to their involvement in relaying signals from the DnaJ-binding site to the ATP-binding site. As to the SBD, T417 and D481 take part in a hinge region that allows for the concerted reorientations of the two domains with respect to each other, while G506 mediates the intradomain movements of the SBD α-helical lid with respect to the β-sandwich; A435, M404 and E430 exhibit strong coevolutionary patterns and serve as *sensors* for binding the substrate; A503-S505 function as *effectors* mediating the communication between the β-sandwich and the α-helical lid of the SBD; and K414, K452, A480 near the interdomain interface are distinguished by their strong coevolution with DnaJ-binding site residues on subdomains IA and IIA. The results provide us with new testable hypotheses concerning the roles of individual residues and correlated mutation sites, some of which (key roles of L177 and T417) are experimentally verified in the current study.

## Results

### Gaussian Network Model identifies residues (T417, D481, G506 and V389-L392) that play key roles in interdomain hinge motions

In order to identify cooperative domain motions, which usually correlate with allosteric changes in conformation, we analyzed the ATP-bound DnaK structure using the GNM. This model approximates the system as a network of harmonically coupled nodes, each node representing a residue, and yields a unique solution for collective modes of motion encoded by the structure. Each mode is characterized by a particular distribution of residue fluctuations away from their equilibrium coordinates, termed the mode mobility profile (see Text S1). Modes at the low frequency end of the mode spectrum are highly collective, i.e. they are large-amplitude cooperative movements of domains being usually distributed across the entire structure, hence their description as *global modes* (as opposed to *local* modes, which involve localized interactions between a few residues). Residues exhibiting minimal displacements in global modes are usually referred to as hinge sites. They are practically fixed in space, serving as hinges at the interface between the domains or substructures that undergo concerted movements. Hinge sites corresponding to global modes are called global hinges. A few global modes are usually sufficient to account for the interdomain or intersubunit couplings that often enable allosteric responses [27], [32]. Residues located at global hinges play a key mechanical role often required to accommodate functional changes in structure, and as such, they tend to be evolutionarily conserved [29].

**Figure 2:**
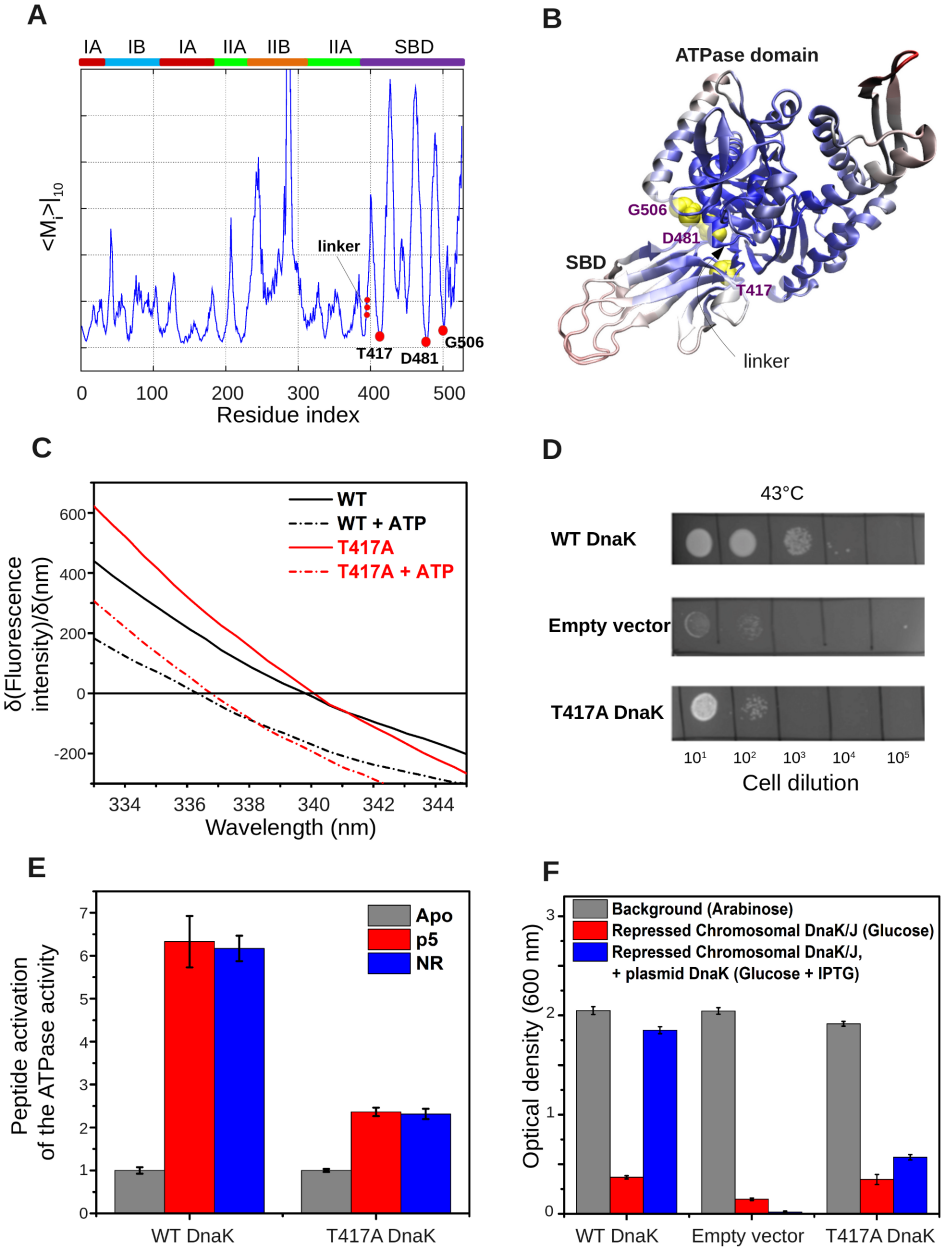
DnaK residues identified from GNM-mobility play a key role in interdomain allostery. (**A**) GNM-predicted mobility profile, <*M_i_*>|*_10_*, evaluated for the ATP-bound structure [25] (**D** in **Figure 1**), residues 4-530. The boxes on the upper abscissa show the residue ranges of subdomains IA, IB, IIA and IIB of the NBD, and the SBD (same color-code as in **Figure 1**). Minima (T417, D481 and G506 in the hinge region) on SBD and linker residues (V389-L392) are marked with red dots. (**B**) Color-coded ribbon diagram based on mobility (*red*: most mobile; *blue*: least mobile). The most mobile region on the ATPase domain (colored *pink-red*) is the NEF-binding subdomain IIB (residues G228-V309). The global mobility is based on the *m* = 10 GNM lowest frequency modes, which account for 40% of the overall dynamics. Three highly mobile C-terminal residues are truncated to permit a clearer visualization. (**C**) ATP-induced fluorescence shift of T417A DnaK variant relative to that of WT DnaK, shown as first derivatives of fluorescence spectra recorded in the absence (solid) and presence (dashed) of 1 mM ATP. Data for WT DnaK are shown in black and those for T417A DnaK in red. The ATP-induced blue shift of the T417A DnaK variant (3.3±0.5 nm) indicates that it adopts a domain-docked conformation in the presence of ATP to an extent comparable to WT DnaK (blue shift 3.5±0.5 nm). (**D**) Since cell growth at elevated temperatures strictly depends upon functional DnaK, the ability to grow after heat shock was used to assess the *in vivo* function of the T417A DnaK variant. Plates are shown that have been incubated at 43 °C after inoculation by serial dilutions of DnaK^–^ *E. coli* cells transformed either with an empty vector or with plasmids encoding the WT or T417A DnaK variant. Despite its ability to undergo the normal ATP-induced conformational rearrangement, T417A DnaK cannot support growth after heat shock. (**E**) The ATPase rates of the T417A DnaK variant relative to WT rates: basal (grey) and upon stimulation by a model peptides (red: p5, CALLLSAPRR, and blue: NR, NRLLLTG). Note that the peptide-induced interdomain allosteric communication responsible for the ATPase stimulation is significantly reduced in T417A DnaK. (**F**) Growth of *E. coli* cells that lack SecB is stringently dependent on functional DnaK [20]. Shown are relative cell densities of cells transformed with either the empty vector or plasmids encoding IPTG-inducible DnaK genes for WT or T417A DnaK variant, either in the presence of arabinose (grey), glucose (red), and glucose + IPTG (blue). Arabinose and glucose induce or repress (respectively) the expression of the chromosomal copies of the *dnaK* and *dnaJ* genes, which have been placed under the control of the P_BAD_ promoter, while IPTG induces expression of the plasmid-encoded DnaK variants. The optical density to which these cultures grow correlates with the degree of *in-vivo* functionality of the expressed DnaK variants.

**Figure 2A** displays the mobility profiles resulting from the cumulative contribution of ten such collective modes evaluated for conformer **D** (a homology model of ATP-bound Hsp70 based on the structure of Hsp110 [25]). Calculations repeated for the ATP-bound structures resolved by Qi et al. [26] and Kityk et al. [25] robustly reproduced the same results (see **Figure S2A**), suggesting that the homology model provides an adequate representation of the overall structure and dynamics of ATP-bound DnaK. Peaks represent the most mobile regions, and minima coincide with residues spatially constrained into functionally required geometries.

Three observations can be made based on the results depicted in **Figure 2A**: First, the profile of the NBD (residues 1-388) is highly similar to that previously obtained for the isolated ATPase domain [23]: Global hinges (minima) are observed at highly conserved sites including the nucleotide-binding region such as G10-T12, G196-G198, G229-D231, the conserved proposed proline switch and its close vicinity (P143-K155) and R167. This close similarity means that the NBD maintains its intrinsic dynamic (modular) character in the ATP-bound Hsp70 conformer. Second, the linker residues V389-L392 (shown by *red dots*) lie in a minimum centered at D385, i.e., the loop region enclosing the linker serves as a hinge that allows for the relative movements of the two domains. Mutations in the hydrophobic linker residues have been indeed observed to severely impair allosteric communication in DnaK [33], [34], and the linker has been reported to play a key role in mediating interdomain coupling [17], [35]. Third, three SBD residues occur at minima in the global mobility profile: T417, D481 and G506. As can be seen in the ATP-bound DnaK structures [25], [26] (**Figure 2B**), T417 and D481 are located at the NBD-SBD interface, positioned to play a role in interdomain communication; whereas G506 acts as a hinge site enabling the reorientation of the α-helical lid of the SBD (relative to the β-sandwich).

The key roles of the interface residues T417, D481 and G506 in the allosteric function of DnaK are supported directly by experimental results. In the case of T417, NMR analysis of an apo form of the DnaK βSBD (the β-subdomain without the helical lid) showed that the loop containing T417 exhibits a large reorientation [36]. As a further test, we mutated T417 to Ala in DnaK and compared its allosteric properties to wild type (**Figure 2C**–**F**). We found that *in vitro* T417A DnaK is partially defective in interdomain communication as a consequence of this relatively minor mutation. Specifically, the two domains of T417A DnaK still dock onto one another in the ATP-bound state as indicated by the characteristic blue shift of W102 fluorescence relative to apo- or ADP-bound DnaK. However, substrate-induced stimulation of the T417A DnaK ATPase rate is more than 2-fold lower than that of wild type DnaK, demonstrating that it is impaired in interdomain allosteric communication. Importantly, the ability of this mutant DnaK to protect cells from heat shock and to support cell growth in a DnaK/SecB deficient strain are markedly reduced relative to wild type, showing that T417 is crucially important for functionality of DnaK *in vivo.* The defect is more severe at high temperature.

Additionally, D481 mutations to L or V in DnaK were found to be highly deleterious, causing defects in *in-vivo* protection against heat shock and in an *in-vitro* disaggregation assay [37]. In recent work, we explored the consequences of mutation of D481 to N [8]. Despite the conservative nature of this mutation, the allosteric equilibrium between domain-docked and undocked states was shifted in D481N DnaK.

A variant of DnaK harboring a mild mutation of G506 (to A) showed slightly impaired function both *in vitro* and *in vivo* [37], most likely because the mechanical role of G506 as a hinge between the βSBD and the α-helical lid was not drastically perturbed. Nonetheless, we and others have found that the region around G506 is very sensitive to mutation. For example, even a very gentle mutation of the adjacent residue S505 (to C) in DnaK led to a loss of *in vivo* function (R. G. Smock and L. M. Gierasch, unpublished results). Likewise, a mutation of L507 to Ala inhibited the transition to the ATP-induced docked conformation and impaired growth in an *in vivo* complementation assay [26].

### PRS analysis identifies two groups of residues, acting as sensors or effectors of allosteric signals

**Figure 3:**
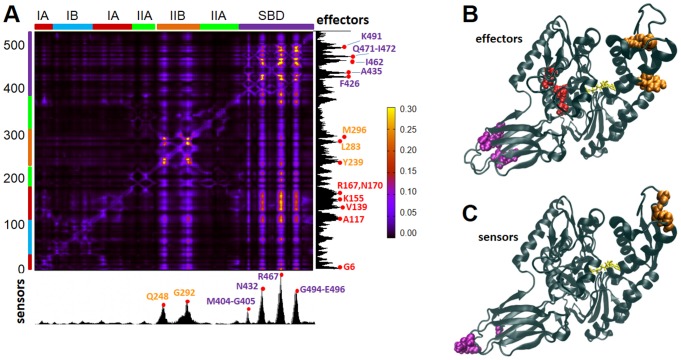
PRS results identify highly influential and sensitive residues that likely propagate allosteric signals in ATP-bound DnaK. (**A**) Perturbation-response map. Strongest perturbation-response sites are shown by the brightest colors (see scale on the *right*). The peaks along the bar plots indicate the effectors (*right ordinate*) and sensors (*lower abscissa*), color-coded (by domain/subdomain types, as in **Figure 1**), displayed by color-coded space-filling representation in the respective panels **B** and **C**.

In order to identify the residues responsible for the long-range transmission of allosteric messages, we applied the PRS method to Hsp70. This method, described in Text S1, probes the response of each residue to a perturbation in every other residue. The results are presented in **Figure 3A** (obtained for the homology model [20], and confirmed with the crystal structures [25], [26] representative of conformer **D**, ATP-bound DnaK, – see **Figure S2B**). The *ik* ^th^ entry of the map represents the response of residue *k* to a unit deformation at residue *i*. The strongest interdomain coupling occurs between V139-R167 on subdomain IA and the distal, solvent-exposed loops of the SBD, indicated by the peaks (*bright spots*).

The PRS map provides information on both the *influence (or effectiveness)* of a given residue in transmitting signals when subjected to a unit perturbation (*rows*), and the *sensitivity* of a given residue to those signals (*columns*). The most influential residues (*effectors*; peaks in the *right ordinate* bar plot; colored by domain/subdomain identity in panel **B**) and the most sensitive residues (*sensors*; peaks in the *lower abscissa* bar plot; panel **C**) form mutually exclusive subsets despite their close proximity on the structure. The effectors are clustered in three regions: near the substrate-binding site of the SBD β-sheet (*purple*), the NEF-binding site (subdomain IIB; *orange*) and the subdomain IA core (G6, A117, V139, K155, R167 and N170) of the NBD (*red*). The former region includes a number of hydrophobic residues (A435, F426, I462, I472), in addition to K491 and Q471. These are proposed to efficiently propagate structural perturbations induced by substrate binding. For example, F426 occupies a central stabilizing position between the two β-sheets, and F426 and I462 are implicated in substrate binding, since an I462T mutation reduced binding affinity and caused loss of function *in vivo* [38], and an F426S mutation abrogated substrate-binding ability in DnaK [39]. The hydrophobic residues in the NBD subdomain IA core that emerge as effectors, on the other hand, presumably transmit signals from the interface to the nucleotide-binding site of the NBD. Their central location and strong influence on both the NEF-binding and substrate-binding sites suggest a role in establishing allosteric communication across the chaperone.

As to sensor residues, six peaks are distinguished, centered at Q248, G292, M404, N432, R467 and G494. These are located at the exposed loops of subdomain IIB and the SBD (**Figure 3C**), where the NEF and substrate bind, respectively. Their sensitivity to perturbations is presumably favored by the overall Hsp70 architecture and permitted by the lack of spatial constraints in their close neighborhood. Notably, R467 forms a salt bridge with the α-helical lid [40] in the ADP-bound state, and its high sensitivity may be a requirement for facilitating the conformational switch between conformers **C** and **D**.

### Subdomain IA of the NBD plays a key role in mediating interdomain allostery via strong influence on interdomain linker

**Figure 4:**
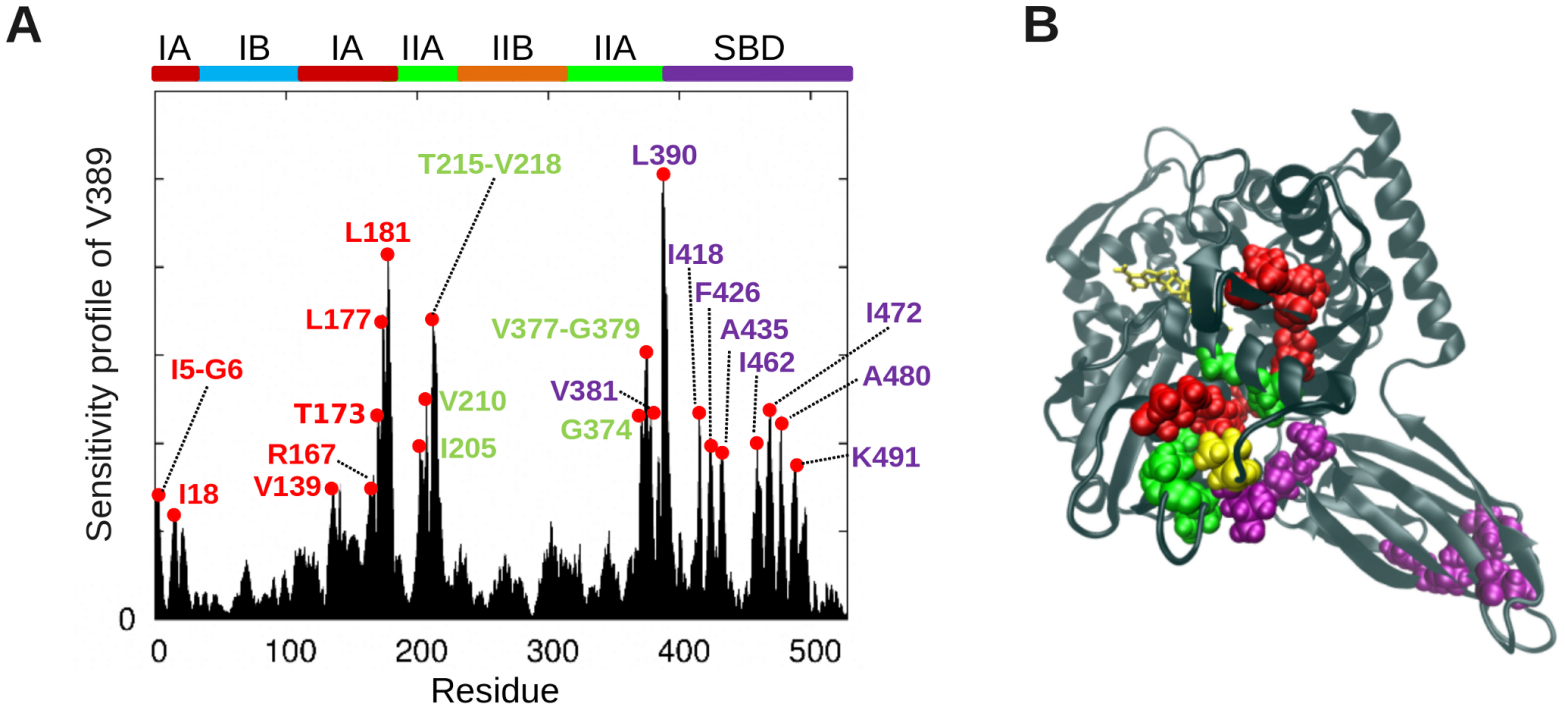
Influence of DnaK residues on the linker residue V389. (**A**) Effectiveness/influence profile with respect to the linker residue V389, obtained from the PRS analysis. Peaks highlight the most influential residues. Labels are colored according to subdomains. (**B**) Location of the most influential residues, shown in *sphere* representation, colored by subdomain/domain id. The perturbed residue, V389, is shown in *yellow* spheres, and the ATP in *yellow* stick representation.

In order to further examine whether the effectors identified above play a key role in interdomain signal transmission, we examined the residues that exert a strong influence on the linker (V389-L392) and hinge site residues D481, G506 and T417 identified above (**Figure 2**). The sensitivity profile of the linker residue V389 (**Figure 4**) confirmed the effector role of subdomain IA and SBD residues identified above. Peaks occur at subdomain IA and SBD residues overlapping with, or closely neighboring, those identified in **Figure 3**.

We additionally note that a number of subdomain IIA residues (V205, V210, T215-V218, Q378-G379; *green*) are strongly coupled to the linker. Their potential role in stimulating ATPase activity will be discussed below, in the context of their interactions with the DnaJ co-chaperone. Calculations repeated for the other three hinge residues (**Figure S3**) showed that all three elicit strong responses at the β-sheet sensor region (panels **A**, **C** and **E**), are highly sensitive to perturbations at subdomain IA (near helix 6 (N147-A161) in particular) (panels **B**, **D** and **F**), in support of their bridging role between substrate-binding site and subdomain IA effectors.

Previous studies have noted that there is a dynamical coupling between the α-helical lid of the SBD and the interdomain linker [40]. Perturbation of D481 and G506 are also noted to elicit a mild response in the α-helical lid, suggesting that those residues might affect the docking of the helical lid onto the NBD. It is worth noting that the α-helical lid contributes to the allosteric interactions [41], but these are still retained in its absence [36], which may explain the weaker response compared to the other highly sensitive sites.

### A network of conserved interactions on subdomain IA assists the hinge region in propagating signals between substrate- and ATP-binding sites

**Figure 5:**
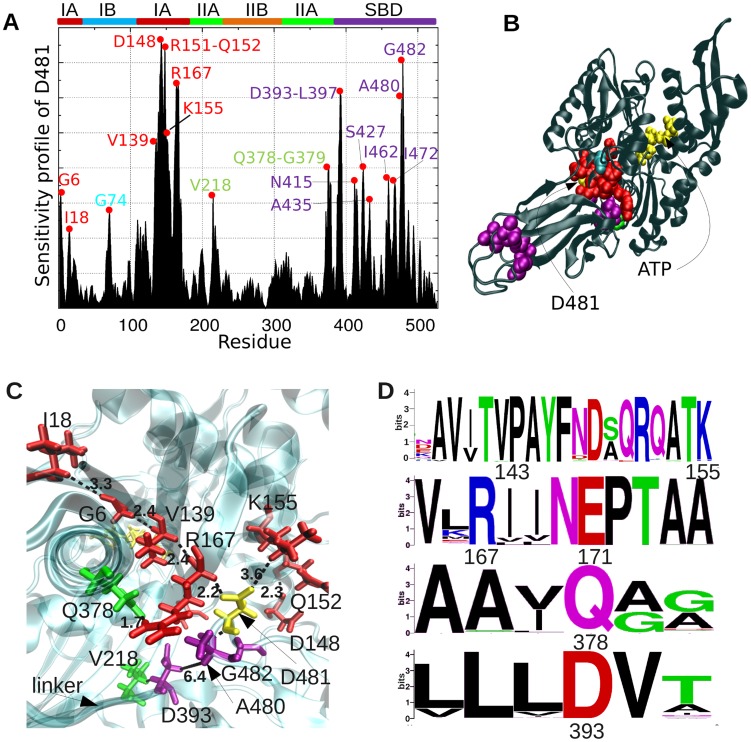
Sensitivity profile of global hinge site, and network of conserved interactions between effector residues at subdomain IA and the linker. (**A**) Sensitivity profile of D481 (representing the hinge region), showing the predominance of effector residues belonging to subdomain IA helix 6 (D148, Q152, K155) and central residues (G6, I18, V139, R167) in the neighboring four strands, and those on β SBD. (**B**) Location of these effectors on DnaK structure. (**C**) Network of interactions between effectors in the neighborhood of D481 (*yellow stick*). Some inter-residue distances are shown as dashed lines; units in Å. ATP is shown in *yellow stick*, in the back. (**D**) Sequence logo plot describing the conservation level of these effectors. Symbol sizes scale with the frequency of different amino acid types at the sequence position.

We performed a more detailed examination of the specific interactions that enable long-range communication, focusing on D481, as a key residue at the global hinge region. **Figure 5A** displays its sensitivity profile in response to perturbations at all residues obtained for the crystal structure. Consistent with **Figures 3** and **S2**, the effectors (peaks) mainly lie in ATPase subdomain IA, on and near helix 6 (N147-A161).

Closer examination of the spatial properties of these effectors reveals a network connecting I18-G6-V139-R167 to D481 via close (<3.5 Å) interatomic interactions (**Figure 5C**). Three charged residues, K155, D148 and D393 (on the linker) complement these effectors. R167, which was also identified by Chiappori et al. [24] to be a significant player in signal transduction, closely interacts with Q378, which further consolidates the network of interactions. Some of these residues (e.g. V139, D148, R167, V218) serve as effectors not only for establishing the communication between the SBD and the NBD, but also for stimulating the ATPase activity of DnaK in response to DnaJ co-chaperone binding, according to previous experimental observations [33], [42]–[44]. Furthermore, residues identified (**Figure 3**) as effectors on βSBD (*purple*) also exhibit strong influence on D481. This finding further confirms their role in conveying allosteric signals from substrate-binding site to the ATPase domain, via the interface. The evolutionary conservation of these residues (**Figure 5D**; see also **Figure S4** and previous work [23]) is in support of their functional role.

### Sequence coevolution analysis highlights residues involved in co-chaperone recognition and their coupling to the NBD-SBD interfacial region

In order to identify patterns of correlated substitutions, which may provide insight into how the allosteric network has been preserved throughout evolution across the Hsp70 family, we performed a thorough analysis of sequence coevolution patterns. In previous work [19], we used an extension of the statistical coupling analysis (SCA) method [45] to simultaneously study the coevolution between structural regions (or groups of correlated amino acids, termed sectors [46] and the functional divergence between family members using a multiple sequence alignment (MSA) that contained sequences from both Hsp70 and Hsp110 subfamilies. In contrast, we focus here on the pairwise coevolution of individual residues, and in order to obtain robust results (i) we use a significantly larger MSA, composed of 2,608 members of the Hsp70 family (see Text S1), (ii) we repeat our calculations with five different methods, and (iii) we identify those pairs of residues confirmed by at least two independent methods to rank in the top 0.06% of all (212 x 388 = 82,256) NBD-SBD residue pairs (rank-ordered by coevolutionary signal strength). The methods we adopted to accomplish this aim are: (1) mutual information MIp with the Average Product Correction [47]; (2) Observed-Minus-Expected-Squared (OMES) covariance [48], proposed for filtering out inaccurate sequence correlations between non-interacting domains or molecules; (3) SCA [45]; (4) direct coupling analysis to obtain direct information (DI), introduced [49], [50] to remove signals originating from indirect interactions; and (5) PSICOV [51], also removing indirect interactions via inversion of sequence covariance matrix.

**Figure 6:**
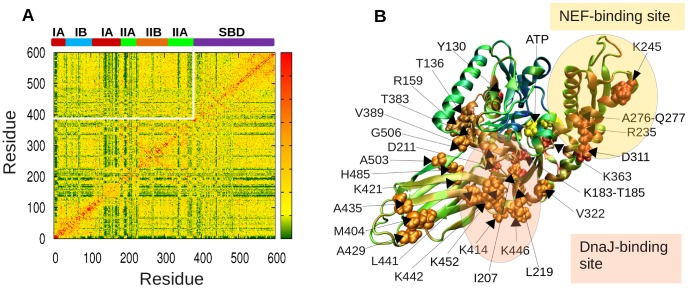
Results from coevolution analysis of Hsp70 family members. On panel **A**, the heat map based on PSICOV covariance predictions is displayed. The white rectangular frame encloses the portion corresponding to interdomain co-variances. Residue pairs distinguished by strongest interdomain signals are listed in **Table 1** and illustrated in **Figure 7**. Those residues exhibiting high cumulative interdomain coevolutionary propensities are labeled and displayed in space-filling representation (labeled on panel **B**) and listed in **Table S1**. The ribbon diagram is color-coded by the propensity of residues to exhibit coevolutionary patterns. NEF- and DnaJ-binding regions are highlighted. The DnaJ region is located mostly on the back of the area shown.

**Figure 6A** shows the coevolution map obtained by PSICOV, where strong signals (*orange-red*) refer to pairs of residues with high coevolution tendencies (and the maps evaluated with the above listed four other sequence covariance analysis methods are presented in **Figure S5**). We focused in particular on the intermolecular segment of the covariance matrix, enclosed in the white upper-left frame in **Figure 6A** (see also counterparts in **Figure S5**), and examined the residues distinguished by strongest *interdomain* coevolution signals. **Table 1** lists the top-ranking pairs deduced from our analysis. Notably, a large number of these signals originate from couplings between subdomain IA or IIA residues (on the NBD) and the interdomain (SBD-NBD) or intradomain (β-sandwich – α-helical lid) hinge/interface residues (on the SBD).

**Table 1:** Strongest coevolution signals between Hsp70 SBD and NBD residues (*).

Pair of residues	Structural position of 1st residue	Structural position of 2nd residue	Remarks
**Pairs making tertiary contacts at interdomain interface**			
E509-R159	SBD internal hinge between β-sandwich and α-helical lid	Subdomain IA	Salt bridge stabilizing the helical lid against subdomain IA in substrate-free low ATPase activity state
A480-L382	SBD interfacial loops at the NBD-SBD global hinge (and interface)	Subdomain IIA	Residue pairs separated by 8 Å on opposite sides of global hinge
I483-K155	SBD interfacial loops at the NBD-SBD global hinge (and interface)	Subdomain IA	Close (<4.5 Å) tertiary contact at the global hinge
K452-A149	SBD β-sandwich end at SBD-NBD interface	Subdomain IA	Close (<4.5 Å) tertiary contact between global hinge and DnaJ binding region
K414-V322	SBD, interdomain interface loop	Subdomain IIA	Close (<4.5 Å) tertiary contact between global hinge and DnaJ binding region
**Neighboring domains, no contacts**			
H422-G379	SBD β-sandwich core	Subdomain IIA near linker	G379 is ∼30 Å away from both H422 and E530 in the open state of the lid
E530-G379	SBD α-helical lid close to subdomain IA	Subdomain IIA near linker	G379 is ∼30 Å away from both H422 and E530 in the open state of the lid
G506-F357	SBD hinge between β-sandwich and lid	Subdomain IIA	F357 is near DnaJ binding site, >30 Å away from hinge site G506
H422-G184	SBD β-sandwich core	Subdomain IA exposed loop	Correlation between spatially distant (>30 Å) pairs
Q471-E217	Effector residue near substrate-binding site	Subdomain IIA	Communication between substrate- and DnaJ-binding sites
**Distant domains**			
L441-Y239, K491-I271	SBD β-sandwich, in close proximity of K452, near interface	Subdomain IIB	Allosteric correlation between NEF binding region, and SBD-NBD interface. Y239 and K491 are effectors. K491 is sensitive to V389

(*)predicted by both PSICOV and DI (or another method) to be among their top-ranking 50 correlated pairs (out of a total of >82,000 combination of residues between the SBD and NBD.)

**Figure 7:**
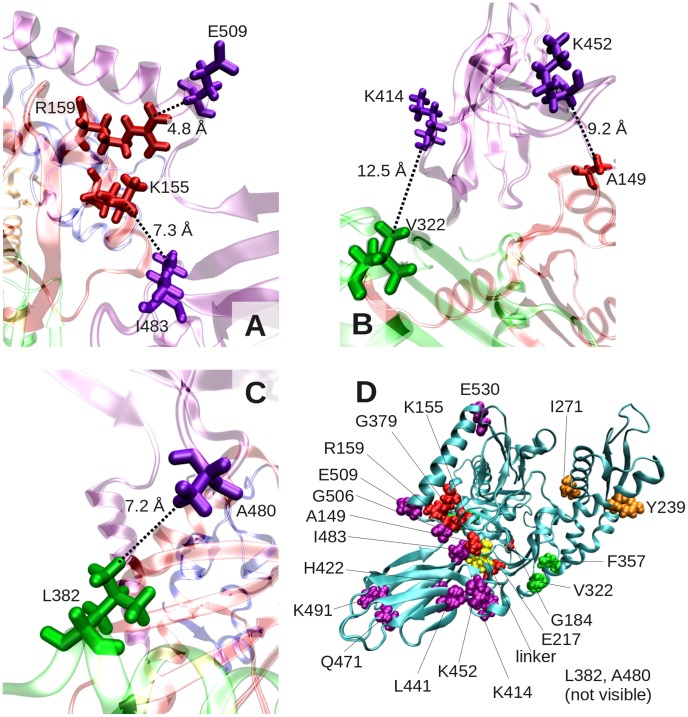
Close-up view of residue pairs distinguished by strong interdomain coevolutionary signals. Panels **A**–**C** display the structural position of residue pairs listed in the first part of **Table 1**, which make tertiary contacts. Panel **D** displays the location of all listed residues on the structure.

Of particular note is the salt-bridge forming pair E509-R159 (the strongest signal in the map), which may be instrumental in docking the α-helical lid against the NBD in the substrate-free, low ATPase activity state of the chaperone (**Figure 7A**). Other pairs making close tertiary contacts and proposed here to be essential to establishing interdomain couplings are listed in the top portion of Table 1, and illustrated in **Figure 7B**–**C** **.** The second group of pairs highlights residues belonging to neighboring domains/subdomains in the 3-dimensional structure, but not making tertiary contacts; and the third involves completely distal pairs between subdomain IIB and SBD effectors near the interface. Previous systematic analysis with large MSAs showed that PSICOV (and DI) are remarkably effective in detecting pairs that make tertiary contacts [51]. The observed correlations between those distant residues may be attributed to their common ancestry, although it is worth noting that they were detected by both PSICOV and DI to be among the strongest signals.

In order to consolidate the results and closely examine the potential involvement of co-chaperone binding sites in strong coevolutionary patterns, we evaluated the *cumulative interdomain coevolution propensity* for each residue, obtained from the sum of the rows/columns of the covariance submatrix associated with intermolecular correlations (see Text S1). The resulting top-ranking residues, organized by subdomains, are listed in **Table S1**, along with the (multiple) covariance analysis methods that support them. **Figure 6B** displays the location of those residues (*space-filling*) in the structure. The structure is color-coded according to these cumulative propensities (*blue*: lowest; *orange-red*: highest). The residues distinguished by strong intermolecular coevolution propensities, highlighted and labeled in **Figure 6B**, can be grouped into clusters of spatially contiguous residues. Two such clusters are on the SBD β-sandwich: one near the substrate-binding site (M404, A429, A435, *sensor* residues), and another at the interface with the NBD (containing several lysines - K414, K421, K442, K446, K452; *effectors*). These co-vary with two clusters on the NBD: one composed of residues (T185, I207, D211, E217) at the interface on subdomain IIA and the linker residue V389 (and its sequential neighbors), extending to K183, Y130 and T136 on subdomain IA (*effectors*); and the second at NEF-binding region, including subdomain IIB (R235, K245, A276, Q277) and subdomain IIA (H226, D311) residues. We note the correspondence with the allosteric sector identified in our previous work [19], except for those residues in subdomain IIB, not present in that sector. This difference is due to the more local perspective and elaborate analysis adopted here, which focuses on individual residues rather than sectors.

A large number of co-evolving residues are confirmed upon examination of cumulative coevolution propensities to be located on the solvent-exposed surface of the ATPase subdomains IIA and IIB implicated in co-chaperone binding. Among those in subdomain IIB, Q277 and A276 form a tight turn between the NBD α-helix 10 (D255-L273) and the succeeding two β-strands that form a β-hairpin. This (helix-turn-hairpin) motif was shown by a comparative analysis of various complexes with NEFs to be the most prominent region involved in NEF recognition and binding, distinguished by both high coevolutionary tendency and high mobility [23] (see also the highest peak in **Figure 2**). The region populated by co-evolving residues on subdomain IIA, on the other hand, coincides with, or closely neighbors, the co-chaperone DnaJ binding site. Mutagenesis and NMR data showed that subdomain IIA residues V210-K214 are affected by DnaJ binding [52], and T215, *E217*, *V218*, T383 and linker *D388-L392* either bind DnaJ or stimulate ATP hydrolysis [53]. The observed coevolution patterns may thus originate from the requirement to bind specific J-domain molecules by different family members, and the requirement to assist in efficiently communicating with nucleotide-binding site and the SBD, which is further supported by the allosteric path analysis presented below.

These results suggest that Hsp70 family members adapt to specific interaction with their cognate co-chaperones by correlated substitutions of amino acids at their co-chaperone recognition sites.

### L177 plays a key role in conveying signals from the linker and DnaJ-binding site to the ATP-binding site

**Figure 8:**
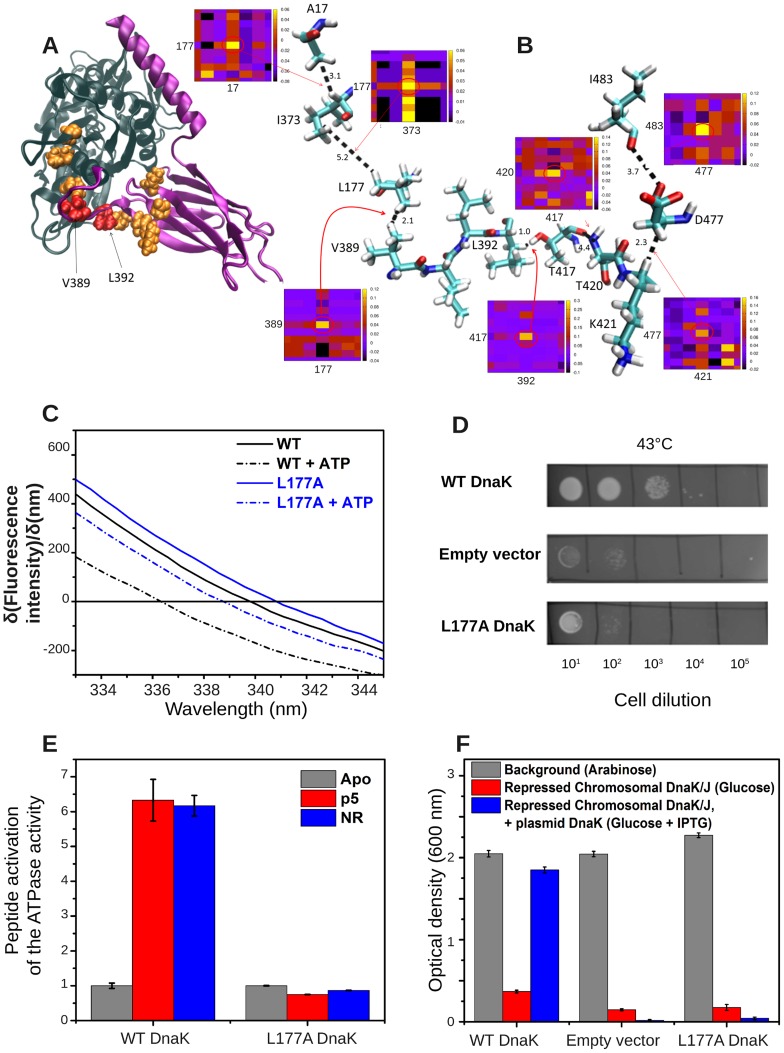
L177 mediates interdomain communication via a cascade of interactions between highly co-evolving residues, including V389 at interdomain linker and T417 and the global hinge. (**A**–**B**) Highly coevolving residues shown in *orange spheres*, except for linker residues V389 and L392, colored *red*. Coevolving pairs of amino acids and their relative spatial positions, shown by stick representation, bridging between the global hinge (T417) and ATP-binding site (via A17). Inter-residue distances are in Å. MIp matrix portions corresponding to these coevolving pairs are indicated. (*bottom*) (**C**) ATP-induced fluorescence shift of DnaK variant L177A relative to that of WT DnaK, as in Figure 2c. The ATP-induced blue shift of the L177A DnaK variant (2.0±0.5 nm) indicates that it is partially impaired in adopting a domain-docked conformation. (**D**) Growth-based functional assay at heat shock temperatures for L177A DnaK, as in Figure 2d. Consistent with the partial impairment of L177A DnaK in domain docking, this DnaK variant cannot support growth after heat shock. (**E**) ATPase rates of L177A DnaK variant relative to WT rates, as in **Figure 2E**. Note that the peptide-induced interdomain allosteric communication responsible for the ATPase stimulation is significantly reduced in L177A DnaK. (**F**) Functional assay for L177A DnaK based on growth in the absence of SecB (as **Figure 2F**), showing that this variant is severely impaired in *in-vivo* function relative to WT DnaK.

To obtain a better assessment of the functional significance of observed coevolution patterns, we focused on the neighborhood of the linker V389-L392, whose interdomain bridging role is recognized to be functional [17], [34], [35]. V389 exhibits a strong co-variance with L177, a hydrophobic residue at the linker-binding pocket (**Figure 8A**–**B**). L177, in turn, is highly correlated with I373, another hydrophobic residue in the vicinity of the linker-binding pocket. Interestingly, both L177 and I373 have been experimentally shown to be highly sensitive to linker binding [35], consistent with the computed coevolution behavior. All amino acids at sequence position 177 on the MSA (F, M, I and L) and almost all at position 373 (98.5% occurrences of A, F, I, L, M, V, W and Y) are hydrophobic. The coevolution of these residues may therefore be a requirement to maintain stabilizing hydrophobic interactions. Furthermore, L177, I373, and the linker have been pointed out to be involved in transmitting signals upon J-domain protein binding [54].

Mutating L177 to A abolishes DnaK *in vivo* activity. *In vitro*, this mutation reduces the DnaK basal ATPase rate, arguing for the importance of this residue to intradomain function, and impairs interdomain communication as assessed by the degree of peptide-induced stimulation of ATPase rate and the ATP-induced blue shift of W102 (**Figure 8C**–**F**).

At the SBD side, we notice the strong coevolution between the linker residue L392 and T417 identified above to take part in the global hinge region (**Figure 2**); both have been pointed out to participate in the sector that mediates interdomain interactions [19]. T417 undergoes correlated mutations with T420, which in turn, correlates with three closely interacting charged residues, K421, D477 and H485, on the β SBD near the interdomain linker.

**Figure 9:**
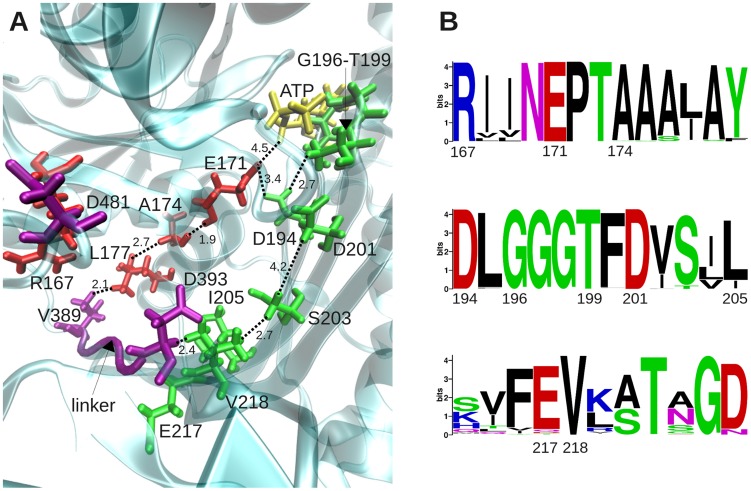
Emerging network of interactions establishing the communication between the DnaJ binding site (near E217 and V389) and the ATP-binding site of DnaK. (**A**) Two interconnected pathways, also coupled to each other (via E171-D194 interaction) are shown, belonging to the respective subdomains IA (*red*) and IIA (*green*) of the DnaK NBD. (**B**) Most on-pathway residues are conserved. L177, which plays a central role is distinguished by its coevolution with V389 (**Figure 8**) and high influence/sensitivity with respect to the majority of displayed residues (**Figure S6**).

A further analysis of the potential role of L177 as an effector (**Figure S5**) indicates its influence on both proximal and distal sites. Proximal sites include V210 and L214 on subdomain IIA, and distal sites include the sensor residues at the far end (substrate-recognition site), and the NEF-recognition site residues D247-Q248-G292 on subdomain IIB. Based on the results presented above, we propose that the network of residues displayed in **Figure 9** underlie the transmission of signals from the DnaJ binding site (near E217 and V389) to the ATP-binding site. The network essentially includes two pathways, predominantly populated by Subdomain IA (*red*) and IIA (*green*) residues, respectively. Subdomain IIA residues V210-K214, and T417 have been pointed out to be affected to different extents by DnaJ binding [52], while earlier studies indicated NBD residues Y146-*D148*, R151, *R167*, N170, *T173*, *T215*, *E217*, *V218*, T383, *D388-L392* to be involved in DnaJ binding or ATP hydrolysis stimulation (see [53]). Several of these residues (those written in italic) are observed here to act as effectors with distinctively high signal propagation effectiveness (see also **Figure 4**). Notably, the predicted mechanism of signal transmission involves two parallel (and closely coupled) pathways, involving a series of conserved residues on subdomains IA (*red*) and IIA (*green*).

## Discussion

We presented here our results from a detailed analysis of the role of different residues in establishing interdomain (SBD-NBD) and intermolecular (chaperone/co-chaperone) allosteric communication in the Hsp70 family of proteins, using a combination of computational and experimental techniques. In order to describe clearly the stages in our analysis and the nature of results we obtain, we have organized in Table 2 the computational results/predictions in three groups: those that are consistent with previous experimental data, and thus validate our computational methods but do not offer new insights; those predicted from our computational methods and tested experimentally in this work, also helping to validate our integrated computational approaches, and lastly novel predictions that will be exciting to test in future experiments, as indicated by the left column. In the last cases, we also included relevant observations from previous experiments that provide indirect support to some of the findings. The table lists residues (*column 2*) distinguished by particular methods (*column 3*) to be exhibit particular roles/properties (hinge, sensor, effector, coevolutionary coupling; *column 4*), as illustrated in corresponding figures (column 5), along with relevant experimental observations (*column 6*) and corresponding references (earlier work or present figure; *column 7*).

**Table 2:** Summary of computational results and relevance to experimental observations.

	Computational Results			Experimental results	
Residues	Method Observation		Ref	Observation	Ref
**Computational data consistent with previous experimental data**					
V389-L392 (linker)	GNM	Linker residues lie in a global hinge site and contribute to establishing interdomain communication	Fig 2A–B	Mutations in the linker residues impair allosteric communication	[17], [33]–[35]
D481 and G506	GNM	Located at minima in global mobility profile, i.e. the global hinge region	Fig 2A–B	D481L(V) and G506A mutations impair DnaK function	[25], [26], [37]
V389, L177, I373	MIp	Coevolution of (V389, L177) and (L177,I373)	Fig 8A–B	L177 and I373 are sensitive to linker binding.	[35]
L177- [V210, L214, V218, D388, L391]	PRS	L177 is coupled to subdomain IIA residues implicated in DnaJ binding	Fig S6 Fig 9	L177 is involved in signal transmission upon DnaJ binding.	[49]
F426, I462	PRS	Effectors at βSBD - transmit signals from substrate-binding site to NBD strong coupling to linker residue V389 and to hinge residue D481	Fig 3A–B Fig 4A Fig 5A	I462T mutant showed reduced binding affinity and loss of function *in vivo,* F426S abrogated substrate-binding ability in DnaK	[38], [39]
**Predicted and validated by current experiments**					
E171, A174, L177, V389	MIp	A network of conserved or coevolving residues connecting ATP-binding site to SBD	Fig 9	L177A abolishes (reduces) DnaK activity *in vivo (in vitro)*.	Fig 7C–F
E171, A174, L177, V389	PRS	Strong sensitivity of linker residues D388-V389 to L177	Figs 4 Fig S6A		
T417	GNM	T417 participates in the hinge site mediating the coupling between NBD and SBD	Fig 2A–B	T417A DnaK is partially defective in interdomain communication *in vitro,* protection from heat shock is reduced *in vivo*	Fig 2C–F
**Computational predictions to be tested/verified**					
G6, A117, V139, D148, K155, R167, N170, E171	PRS	Effectors in subdomain IA for information transfer between ATP-and substrate-binding sites,strong coupling to linker V389-D393 and to hinge residue D481	Fig 3A–B Fig 4A Fig 5A	V139, D148, R167 and V218 stimulate ATPase activity in response to DnaJ. The role of others is to be verified	[33], [42]–[44]
A435, Q471, I472, K491	PRS	Other effectors (in βSBD) of signals from substrate-binding site to NBD sub IA and IIB; Strong coupling to linker (V389) strong coupling to hinge (D481)	Fig 3A–B Fig 4A Fig 5A		
M404-G405, N432, R467, G494, E496	PRS	Sensor residues near substrate-binding site of the SBD	Fig 3A,C	R467 forms a salt bridge with the α-helical lid in the ADP-bound state	[40]
I207, D211, L219, H226, S307, D311, V322, V331, K363, T383	PSICOVDI, MIp, SCA, OMES	NBD Subdomain IIA residues distinguished by strong co-evoutionary propensities with respect to SBD residues	Table S1 Fig 6	V210-T215, E217, V218, T383 are among residues reported to be implicated in DnaJ binding.	[52], [54]
D129, Y130, P134, T136, E17, R159, K183-T185	PSICOVDI, MIp, SCA, OMES	NBD Subdomain IA residues distinguished by strong co-evoutionary propensities with respect to SBD residues	Table S1 Fig 6		
S234, R235, K245, P256, A276	PSICOVDI, MIp, SCA, OMES	Same as above, for subdomain IIB	Table S1 Fig 6		
R159-E506	PSICOVDI, MIp, SCA, OMES	Strongest coevolution signal; interdomain salt bridge, potentially stabilizing the α- helical lid in the low ATPase activity state of DnaK	Table 1* Fig 7A–C		
L382- A480 K155- I482 A149- K452 V322- K414	PSICOVDI, MIp, SCA, OMES	Strong coevolution signals between residues belonging to neighboring domains, suggestive of a role in maintaining stability or allostery	Table 1* Fig 7A–C		
Q248, G292	PRS	SBD sensors near NEF-binding site	Fig 3A,C		
Y239, L283, M296	PRS	SBD effectors near NEF-binding site	Fig 3A,B		

(*) See also the distant pairs that exhibit strong coevolution signals, listed in *Table 1* *, and labeled in* *Fig 7D* *.*

The coupling between the linker-binding site and the nucleotide-binding site of Hsp70 has been noted in previous studies [17], [35], and the present analysis highlights the sensors and effectors that establish this coupling. Our analysis consistently pointed to the important role of residues in NBD subdomain IA in establishing this allosteric communication. Among residues that appear to make a dominant contribution to signal propagation, we noticed highly conserved pairs that form salt bridges (global hinge D481 with R167 and also with K155), in addition to small or hydrophobic residues (e.g., G6, I18, V139, A149), which form tight contacts and enable efficient signal transmission (**Figure 5**). Experimentally, we found that a minor mutation in subdomain IA residue L177, such as L177A, abolishes interdomain communication (**Figure 8C**–**F**). The same is true for the hinge residue T417, whose mutation into an alanine critically impairs the allosteric communication between the two domains (**Figure 2C**–**F**).

It is interesting to note that subdomain IA residues not only help establish allosteric communication between the substrate- and ATP-binding sites, but emerge as effectors of the signals conveyed by the co-chaperone DnaJ to the ATP-binding site. Notably, co-chaperone recognition involves a number of co-evolving (sensor) residues near/at the linker (including V389, D211 and E217). The stimulation of ATPase activity, on the other hand, involves a robust network of effectors (**Figure 9**) composed of two interconnected paths: one supported by conserved interactions in subdomain IA (involving A174 and E171, the latter also involved in the stabilization of a hydrogen bond network [24]), and the other by conserved residues in subdomain IIA (V218, S203, D201, D194, G196, T199). The identity of DnaK residues that are involved in, or affected by, DnaJ binding has been recently debated [52], [53]. Our examination highlights the key roles of D148, R167, T173, D211, E217-L219, T383, D388-L392 among those experimentally detected, in addition to A149, L177, F357 and T417 (see also **Tables 1** **and** **2**).

Previous studies invited attention to the involvement of exposed, conserved, polar and charged residues in substrate binding [55], [56]. Our previous work suggests that while coordinating residues at the substrate-binding site are usually conserved, those at recognition sites may undergo correlated mutations to maintain a balance between substrate specificity and structural adaptability [29]. The preponderance of co-evolving amino acids in subdomain IIA and near the linker is attributed here to the adaptability to specific DnaJ recognition, in the same way as the NEF-recognition site in subdomain IIB residues were reported to undergo correlated substitutions [23].

Our results point to a number of key residues in the ATPase domain that propagate the interfacial perturbation to the nucleotide-binding site. Sequence analysis also indicates specific interactions in this region. Certain secondary structure elements are found to mediate distant communication in the ATPase domain. For instance, upon perturbation of the interfacial residues (e.g., D481), helix 10 couples subdomains IA and IIB; and helix 6, in subdomain IA, mediates the coupling of the SBD with the nucleotide-binding site (**Figure S3**). In the SBD, it is interesting to observe that the interfacial perturbations propagate all the way to the exposed end of the β-sandwich (**Figures 3**–**5** **, S3** and **S6**). Sequence coevolution patterns among residues in β-strands β3, β5-β7 disclose tight interactions that may be important to maintaining the long-range coupling. As already mentioned, we found residues L177 and T417 to critically affect interdomain communication upon mutation to alanine, in support of the significance of the coevolution pattern shown in **Figure 8A**–**B**.

The perturbation-response heat map (**Figure 3A**) is not symmetrical, i.e. it has directionality. This property permitted us to distinguish between signal-receiving and -transmitting properties of residues, and identify those residues acting as sensors or effectors. In contrast, conventional correlation analysis based on the fluctuations covariance matrix (see for e.g. [57] would not distinguish between such roles of residues as the covariance matrix is symmetrical. In this respect, PRS emerges as a useful tool for probing the signal transduction properties in allosteric proteins. We note that perturbation-response analyses based on network models have been performed in previous studies as well (e.g. evaluation of commute/hit times in structures modeled as Markovian networks [58], [59], or examination of the change in collective dynamics upon changing the force constants of the springs surrounding a given residue [60]). The approach of Thirumalai and coworkers [60] might be particularly suitable for examining the effect of a local perturbation (an amino acid substitution) on the network; whereas the PRS method provides a metric of the overall signal sensing and propagation properties directly based on linear response theory (see Text S1). The present application shows how sensors are involved in substrate or co-chaperone recognition, and they tend to co-evolve. Their coevolutionary propensities originate from the necessity to adapt to a diversity of co-chaperones. Effectors, on the other hand, play a key role in relaying binding effects to functional sites. Notably, linker residues are unique, as they play a dual role, serving both as sensors (near the DnaJ-binding region) and effectors (contributing to establishing both interdomain coupling and that between the DnaJ- and ATP-binding sites).

The identification of residues acting as sensors and effectors, coupled with coevolution analysis appears to be a promising approach for assessing potential signal transduction mechanisms and generating hypotheses testable by mutational analyses in allosteric proteins. Our study shows that generic functions such as the SBD-NBD allosteric modulation are predominantly accomplished via conserved residues (**Figure 5**), while those associated with co-chaperone activities are transduced by either conserved or co-evolving residues (**Figure 9**). Of particular interest would be to experimentally verify not only the disruption of co-chaperoning function upon mutating on-pathway key residues, but also the restoration of the function by compensating mutations between co-evolving pairs.

## Methods

### *In vivo* functional assays

The heat shock assay was preformed as described previously [61]. The ΔSecB assay was performed as previously described [20].

### Purification of proteins

WT, L177A and T417A DnaK variants were expressed from the pms119-DnaK vector in BB1553 cells and were purified as previously described{Montgomery, 1999 53/id}. Pure DnaK was concentrated, buffer exchanged to remove unbound nucleotide, unfolded in 8 M urea to remove remaining bound nucleotide, refolded into a 10-fold volumetric excess of 10 mM KPO_4_ 100 mM KCl 1 mM EDTA pH 7.6 (PEK) buffer, buffer exchanged eight times into PEK to remove urea, and then buffer exchanged into 10 mM HEPES 100 mM KCl 5 mM MgCl_2_ pH 7.6 (HMK) buffer in a Centricon-30 concentrator (Amicon) prior to flash freezing with liquid nitrogen and storage at −80 °C. Protein concentrations were determined spectroscopically using an extinction coefficient of 𝛆_280_ = 15.8×10^3^ M^−1^cm^−1^ [62]for all the DnaK variants.

### Fluorescence assay

Fluorescence spectra were collected in HMK buffer using a Photon Technology International Alpha Scan Fluorometer (Birmingham, NJ) as described previously [39] for 10 µM DnaK wild type, DnaK L177A, and DnaK T417A in the absence and presence 1 mM ATP. The excitation wavelength was set to 295 nm, and the excitation and emission slits were set to 3 nm and 1 nm respectively.

### ATPase measurements

ATPase rates for DnaK wild type, DnaK L177A, and DnaK T417A were measured using the enzyme-coupled assay previously described [19], [39] using a Biotek Synergy2 microplate reader. One or 10 µM DnaK was used for the peptide-stimulated and basal rate measurements, respectively.

### Structural data

In order to examine the allosteric interactions between the two domains of Hsp70, we utilized the homology model of DnaK, the *E. coli* homolog of Hsp70 [19], where the two domains are in close contact (ATP-bound state). We also repeated our calculations for the structures recently resolved for ATP-bound DnaK —PDB codes 4B9Q and 4JNE [25], [26]— which showed that GNM and PRS results are insensitive to structural details and closely reproduced using these structures (see Text S1 for comparative results).

### Sequence analysis

We evaluated the sequence conservation and coevolution properties of DnaK, starting the MSA retrieved from Pfam for Hsp70 family members (Pfam id: PF00012 [63]). Conservation properties were evaluated using Shannon entropy, and sequence covariance, using the average-product-corrected mutual information (MIp) [47], OMES [48], DI [49], [50], and PSICOV [51] (see Text S1 for more information).

### Perturbation Response Scanning

PRS [28] allows for a quantitative assessment of the influence/sensitivity of each residue with respect to each other. Results are described by NxN heat maps (for a protein of N residues). Row and column averages provide information on the propensity of a given residue to act as a sensor or effector, as explained in more detail in Text S1.

### Gaussian Network Model

GNM is used for evaluating the *mobility profile M_i_* ^(*k*)^ as a function of residue index *i*, for the normal mode *k,* following the protocol described in our previous work [30], [64]. A cutoff distance of 7.3 Å has been adopted for constructing the network connectivity/Kirchhoff matrix **Γ** (see Text S1 for more information).

## Supporting Information

Figure S1
**PRS protocol.** A force with random direction and unit magnitude is exerted on node *i*, and the displacement vector, Δ**R** *^(i)^*  =  (Δ*r*_1x_ *^(i)^* Δ*r_1y_^(i)^* Δ*r_1z_^(i)^* … Δ*r_Nz_* ^(i)^), elicited in all C^α^-atoms is computed. The response of residue *k* is expressed by the square displacement 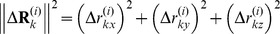. The procedure is repeated *m* times to eliminate potential biases from the direction of the applied force. The resulting average response of the *k* ^th^ residue is 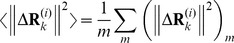.(TIF)

Figure S2 **Comparison of the collective dynamics obtained for the DnaK homology model (HM) proposed by Smock et al and the PDB structures 4B9Q and 4JNE, resolved by X-ray crystallography.** (A) The first and second GNM normal modes computed for HM (*red* curve), 4B9Q (*green*) and 4JNE (*blue*). (B) Sensitivity/influence maps obtained by PRS resulting for the HM, and for the crystal structures 4B9Q and 4JNE.
(TIF)

Figure S3 **Response of the overall structure to perturbations at interdomain hinge site residues T417, D481 and G506 on the SBD.** Panels A, C and E are colored-coded by the response profiles triggered upon perturbing the three respective residues (labeled and shown in yellow sphere), and the lower panels display the sensitivity profile of these three residues upon perturbation of the rest of the structure. Exposed loop residues at the distal end the SBD β-sandwich exhibit the strongest sensitivity (upper panels), while subdomain IA core residues including in particular helix 6 and its close vicinity are the most influential (effector) sites (lower panels).
(TIF)

Figure S4 **Conservation profile of DnaK.** Shannon entropy of DnaK in terms of residue number.
(TIF)

Figure S5 **Results from coevolution analysis.** Upper five maps refer to the sequence covariance between all residues in the two domains, NBD and SBD, of Hs70 family proteins, evaluated by five different methods (labeled). The lower five maps magnify the portions corresponding to intermolecular portions of the maps.
(TIF)

Figure S6 **Influence and sensitivity profiles of residue L177.** (**A**) Influence profile of L177 (*upper panel*) and ribbon diagram, color-coded by the profile. (**B**) Sensitivity profile and corresponding ribbon diagram.
(TIF)

Table S1 **Hsp70 residues distinguished by their strong co-evolution tendencies.**
(DOCX)

Text S1 **Computational and experimental methods, comparison of a homology model to crystal structures, and more results on sequence conservation, co-evolution analysis and PRS.**
(DOCX)
