# Supplementary figures and images for: ATPase Subdomain IA Is a Mediator of Interdomain Allostery in Hsp70 Molecular Chaperones
Source: PLoS Comput Biol. 2014 May 15;10(5):e1003624. doi: 10.1371/journal.pcbi.1003624 (PMC4022485; doi:10.1371/journal.pcbi.1003624)

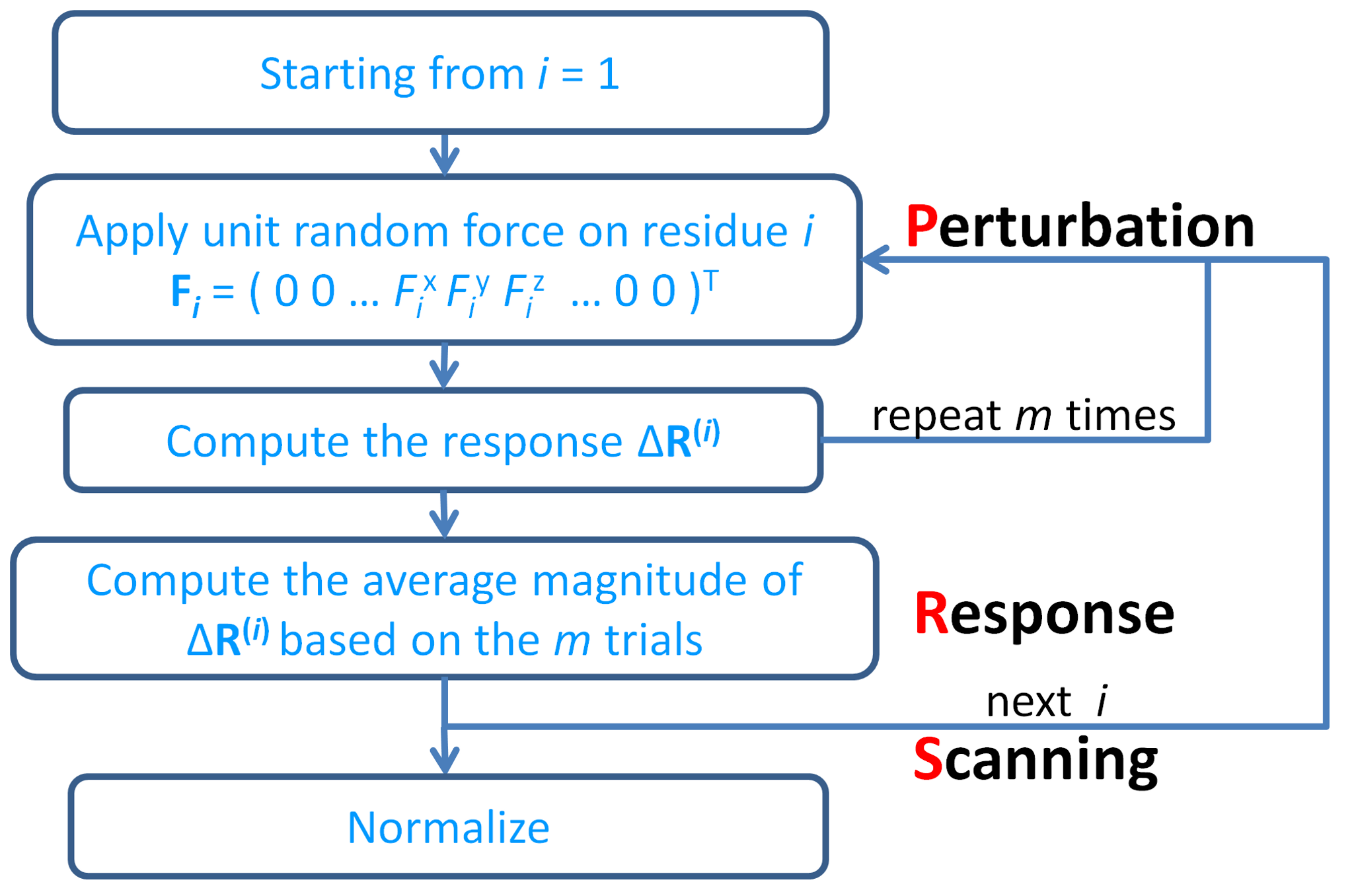

Supplement: Figure S1 — PRS protocol. A force with random direction and unit magnitude is exerted on node i, and the displacement vector, ΔR (i) = (Δr 1x (i) Δr1y(i) Δr1z(i) … ΔrNz (i)), elicited in all Cα-atoms is computed. The response of residue k is expressed by the square displacement . The procedure is repeated m times to eliminate potential biases from the direction of the applied force. The resulting average response of the k th residue is . (TIF) [file pcbi.1003624.s001.tif]

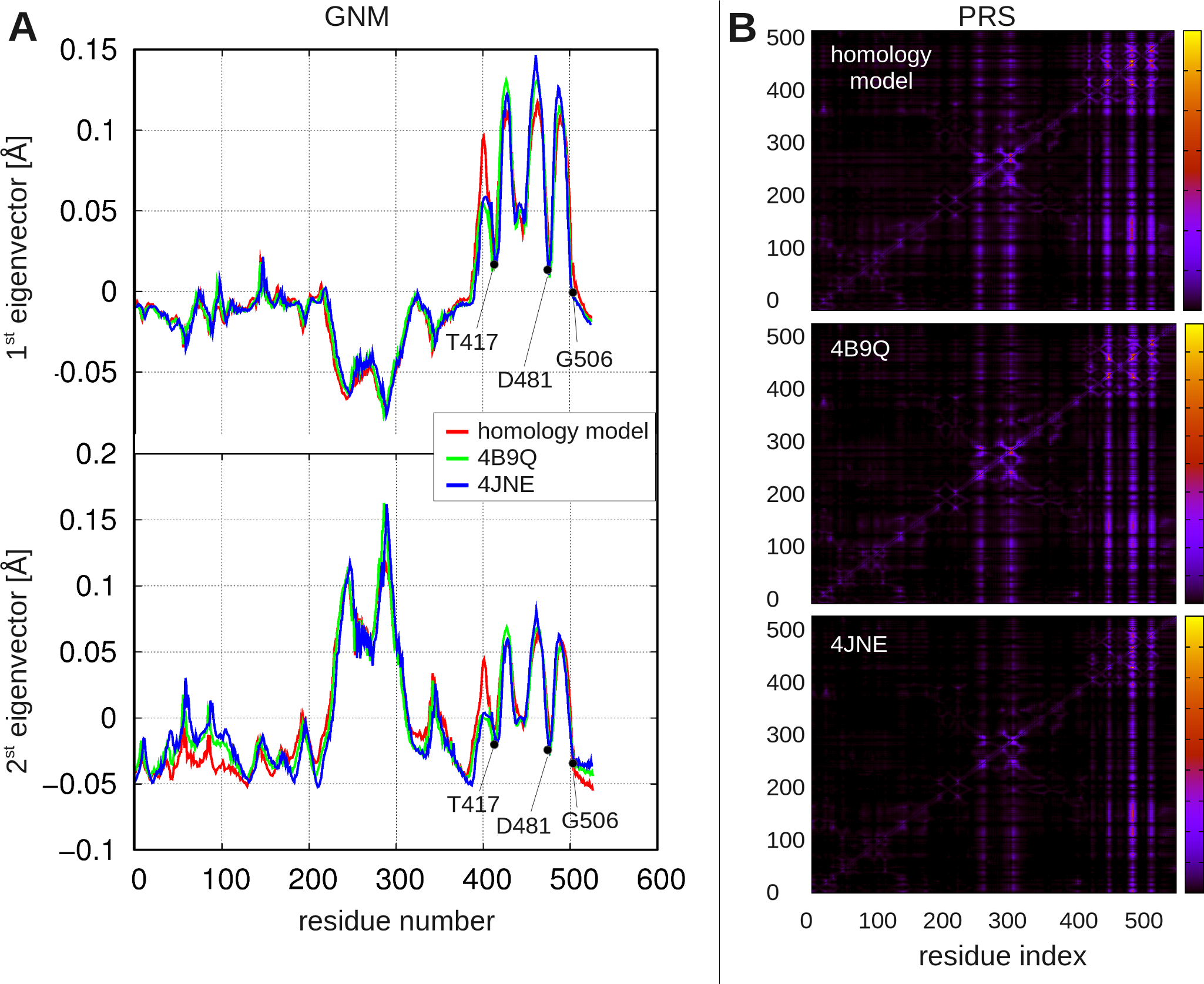

Supplement: Figure S2 — Comparison of the collective dynamics obtained for the DnaK homology model (HM) proposed by Smock et al and the PDB structures 4B9Q and 4JNE, resolved by X-ray crystallography. (A) The first and second GNM normal modes computed for HM (red curve), 4B9Q (green) and 4JNE (blue). (B) Sensitivity/influence maps obtained by PRS resulting for the HM, and for the crystal structures 4B9Q and 4JNE. (TIF) [file pcbi.1003624.s002.tif]

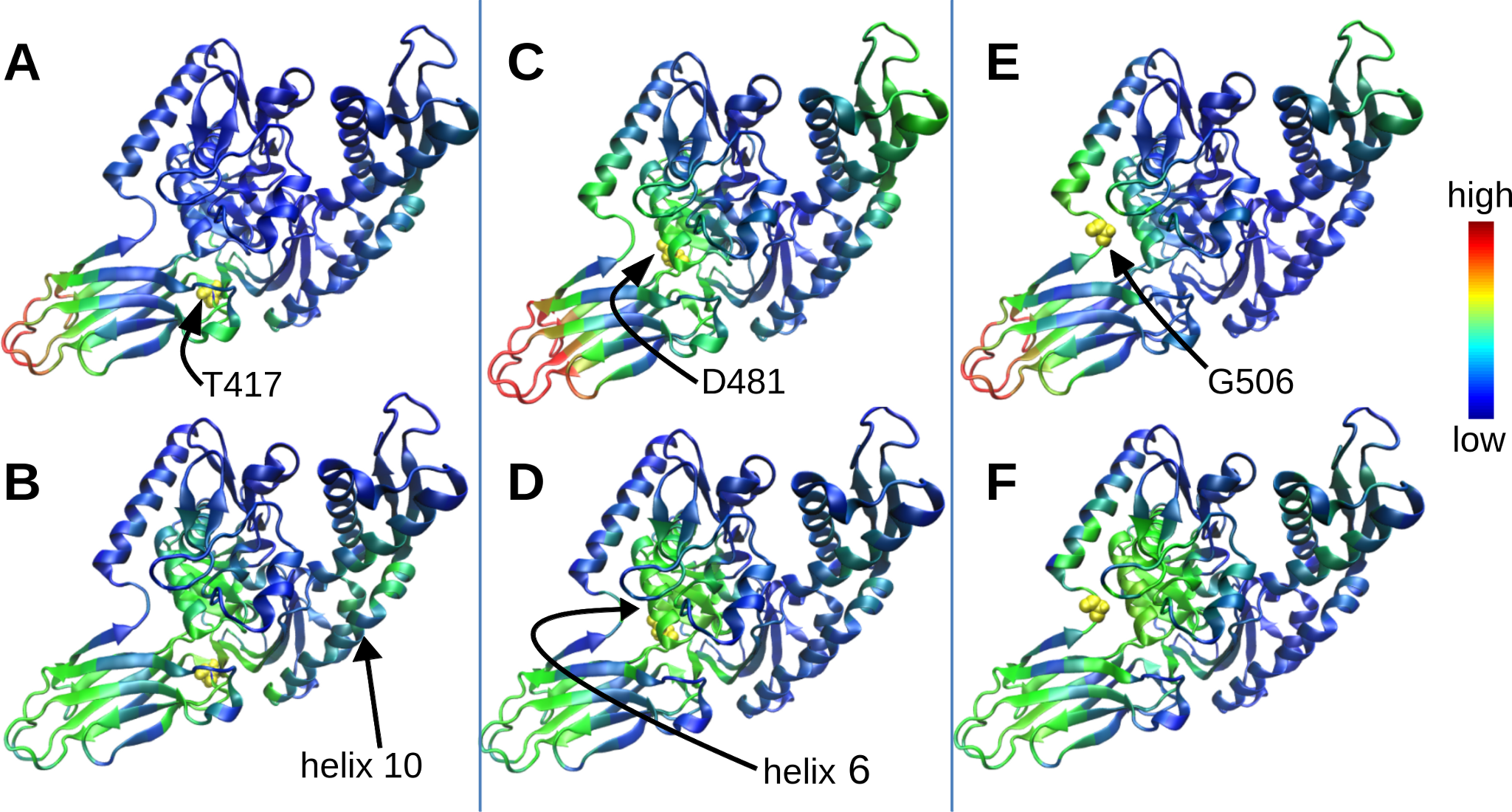

Supplement: Figure S3 — Response of the overall structure to perturbations at interdomain hinge site residues T417, D481 and G506 on the SBD. Panels A, C and E are colored-coded by the response profiles triggered upon perturbing the three respective residues (labeled and shown in yellow sphere), and the lower panels display the sensitivity profile of these three residues upon perturbation of the rest of the structure. Exposed loop residues at the distal end the SBD β-sandwich exhibit the strongest sensitivity (upper panels), while subdomain IA core residues including in particular helix 6 and its close vicinity are the most influential (effector) sites (lower panels). (TIF) [file pcbi.1003624.s003.tif]

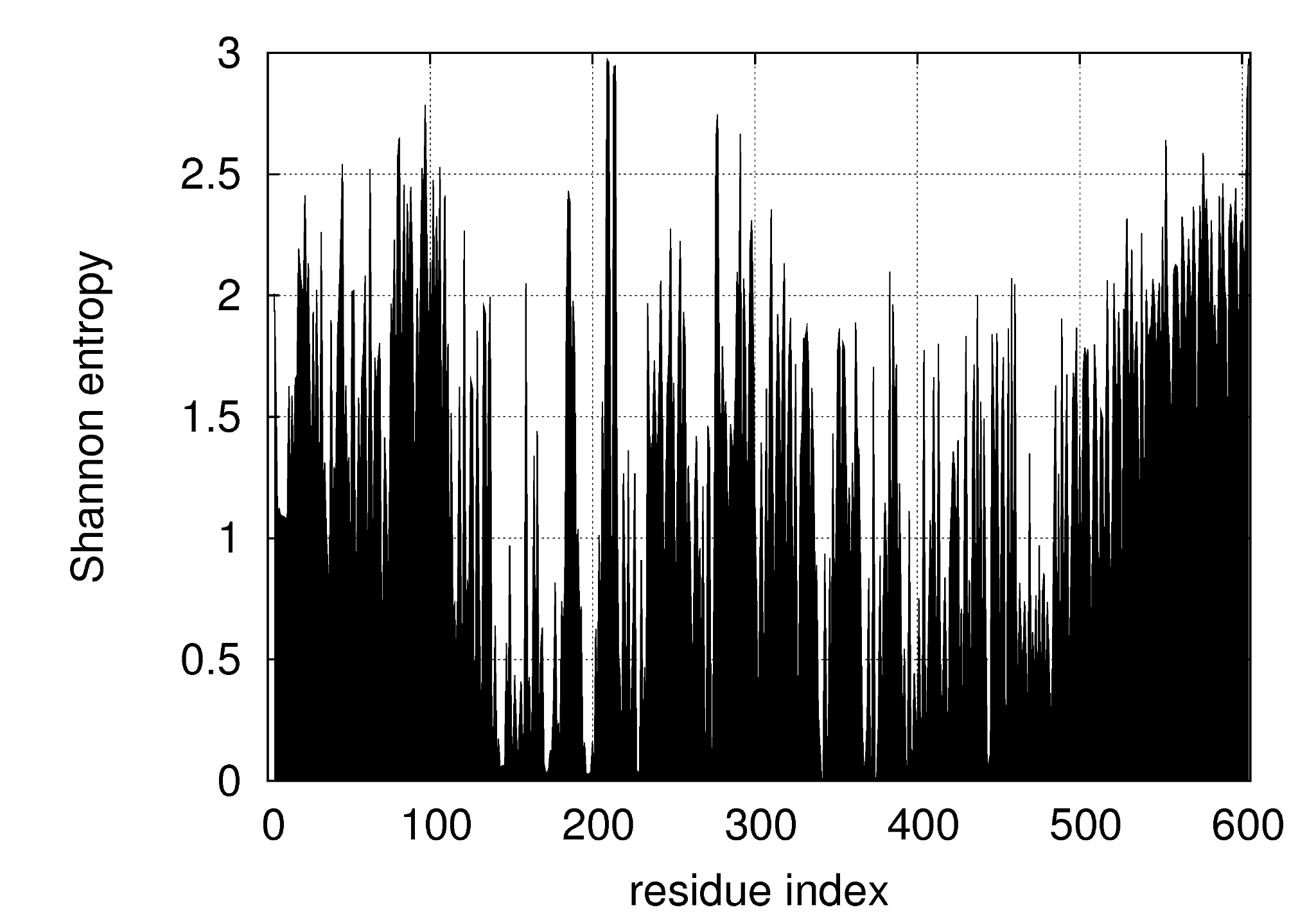

Supplement: Figure S4 — Conservation profile of DnaK. Shannon entropy of DnaK in terms of residue number. (TIF) [file pcbi.1003624.s004.tif]

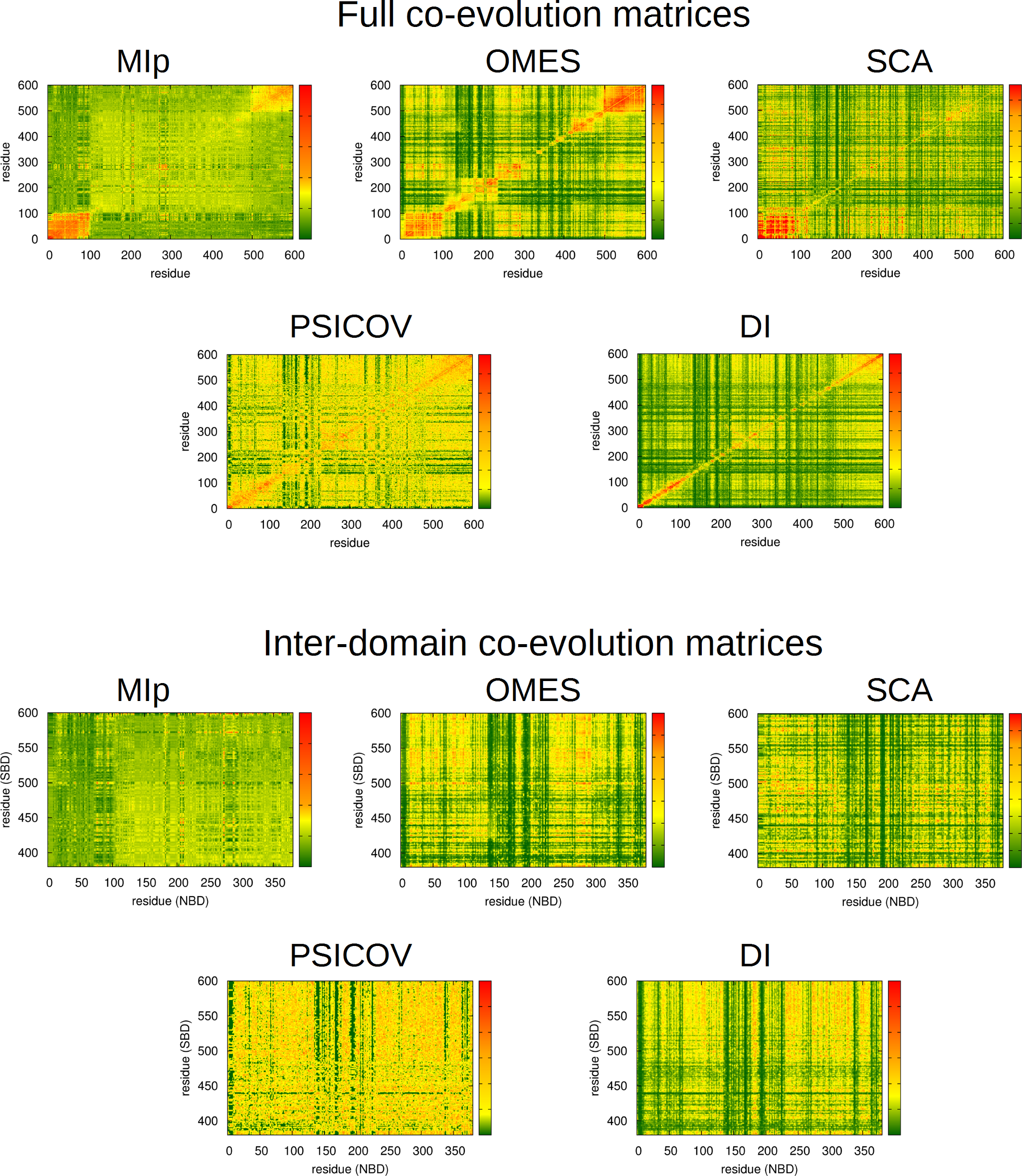

Supplement: Figure S5 — Results from coevolution analysis. Upper five maps refer to the sequence covariance between all residues in the two domains, NBD and SBD, of Hs70 family proteins, evaluated by five different methods (labeled). The lower five maps magnify the portions corresponding to intermolecular portions of the maps. (TIF) [file pcbi.1003624.s005.tif]

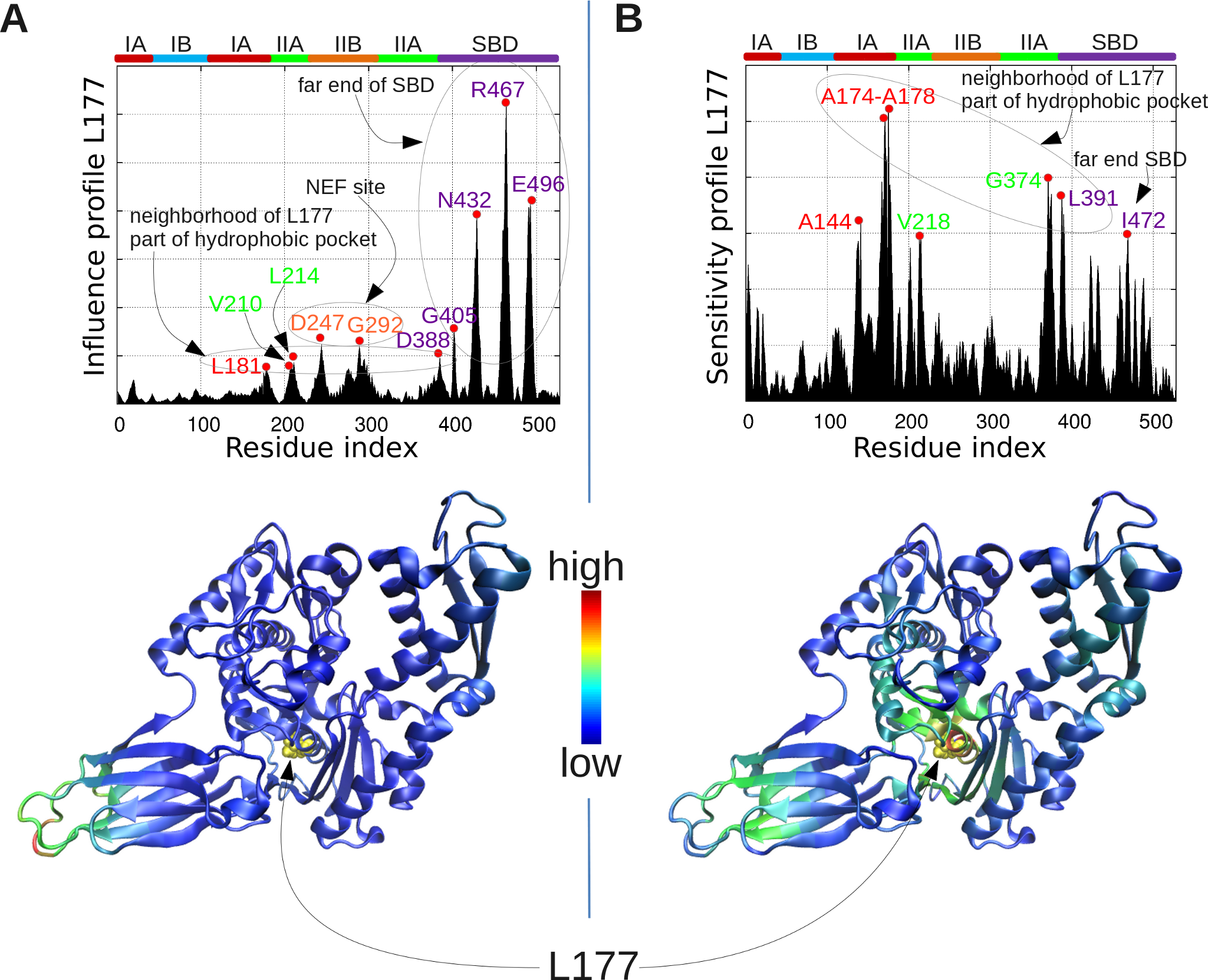

Supplement: Figure S6 — Influence and sensitivity profiles of residue L177. (A) Influence profile of L177 (upper panel) and ribbon diagram, color-coded by the profile. (B) Sensitivity profile and corresponding ribbon diagram. (TIF) [file pcbi.1003624.s006.tif]
