# Supplementary material for: ATPase Subdomain IA Is a Mediator of Interdomain Allostery in Hsp70 Molecular Chaperones
Source: PLoS Comput Biol. 2014 May 15;10(5):e1003624. doi: 10.1371/journal.pcbi.1003624 (PMC4022485; doi:10.1371/journal.pcbi.1003624)
Supplement: Table S1 — Hsp70 residues distinguished by their strong co-evolution tendencies. (DOCX) [file pcbi.1003624.s007.docx]

**Table S1. Hsp70 Residues Distinguished by their Strong Co-evolution Tendencies***

| **NBD** | | **SBD** | | |  |
| --- | --- | --- | --- | --- | --- |
| **Residue** | **Supporting methods** | **Residue** | | **Supporting methods** |  |
| **IA** | |  | | | |
| D129,Y130 | PSICOV, MIp, OMES, SCA, DI | D385 | PSICOV, DI, MIp, OMES | | |
| P134 | PSICOV, DI | V389 | PSICOV, MIp, OMES | | |
| T136,E137 | PSICOV, MIp, OMES, DI | M404 | MIp, PSICOV, OMES | | |
| R159 | PSICOV, DI | A413 | PSICOV, DI | | |
| K183 | PSICOV, MIp, SCA | K421 | PSICOV, SCA | | |
| G184 | PSICOV, DI | A429 | MIp, OMES | | |
| T185 | PSICOV, DI, MIp | M408 | PSICOV, SCA | | |
| **IIA** | | K414 | MIp, PSICOV, SCA | | |
| I207 | PSICOV, SCA | E430 | PSICOV, MIp, OMES | | |
| D211 | PSICOV, MIp | A435 | DI, MIp, OMES, PSICOV | | |
| L219 | PSICOV, DI | T437 | PSICOV, DI, MIp, OMES | | |
| H226 | PSICOV, SCA | L441 | MIp, PSICOV | | |
| S307 | PSICOV, SCAI | Q442 | MIp, SCA | | |
| D311 | MIp, SCA | K446 | MIp, PSICOV, DI, OMES | | |
| V322 | PSICOV, DI | K452 | MIp, PSICOV, OMES | | |
| V331 | DI, SCA | N458 | PSICOV, MIp | | |
| R362,K363 | PSICOV, MIp, OMES | H485 | PSICOV, DI | | |
| T383 | PSICOV, DI, MIp, OMES | A503 | PSICOV, SCA | | |
| **IIB** | | G506 | PSICOV, SCA | | |
| S234,R235 | MIp, DI, OMES |  |  | | |
| K245 | MIp, OMES |  |  | | |
| P256 | PSICOV, DI |  |  | | |
| A276 | MIp, OMES |  |  | | |

**sums of rows or columns of the interdomain portion of co-evolution matrices*
