# Supplementary material for: ATPase Subdomain IA Is a Mediator of Interdomain Allostery in Hsp70 Molecular Chaperones
Source: PLoS Comput Biol. 2014 May 15;10(5):e1003624. doi: 10.1371/journal.pcbi.1003624 (PMC4022485; doi:10.1371/journal.pcbi.1003624)
Supplement: Text S1 — Computational and experimental methods, comparison of a homology model to crystal structures, and more results on sequence conservation, co-evolution analysis and PRS. (DOCX) [file pcbi.1003624.s008.docx]

***Supplementary Information***

for

**ATPase subdomain IA is a mediator of interdomain allostery in Hsp70 molecular chaperones**

**Ignacio J. General^1^, Ying Liu ^1^, Mandy E. Blackburn^2^, Wenzhi Mao^1,3^, Lila M. Gierasch^2,4^ and Ivet Bahar^1, *^**

**^1^***Department of Computational and Systems Biology, School of Medicine, University of Pittsburgh, Pittsburgh, Pennsylvania, USA,* **^2^***Department of Biochemistry & Molecular Biology, University of Massachusetts Amherst, Amherst, Massachusetts, USA,* **^3^***Department of Pharmacology, Tsinghua University, Beijing, China.* **^4^***Department of Chemistry, University of Massachusetts Amherst, Amherst, Massachusetts, USA.*

**Supplemental Information on Methods**

**Computational methods**

**Sequence conservation and coevolution analyses:** We evaluated the sequence conservation and co-evolution properties of the full-length DnaK, starting from a multiple sequence alignment (MSA) of 16,179 sequences (and *N* = 601 columns corresponding to DnaK residues 4-604) based on Pfam data for Hsp70 family members (Pfam id: PF00012, Pfam version 24.0[1]). The MSA was refined in three steps: (i) a reference sequence was identified by screening DnaK wild type sequence [2] against all in the Pfam MSA using the Smith-Waterman algorithm [3]; (ii) the MSA columns that correspond to the reference sequence residues were retained, and (iii) the resulting MSA was then subjected to further refinement, including removal of the redundant sequences using a threshold of 99%, and eliminating the sequences that had more than 20% gaps. This led to an ensemble of 2,608 sequences for evaluating two properties: (a) conservation profile based on Shannon entropies S(*i*) = -∑*_xi_ P*(*x_i_*) ln *P*(*x_i_*) at each position *i*, where *P*(*x_i_*) represents the fraction of amino acid of type *x* at the sequence position *i*. The summation is performed from *x_i_* = 1 to 21, where insertion/gaps are considered as the 21^st^ type (see resulting profile in **Fig S4**); and (b) coevolution propensities based on 4 different methods: Mutual Information with the APC correction (MIp)[4], the Observed-Minus-Expected-Squared (OMES) covariance algorithm[5], Direct Information (DI)[6,7], and PSICOV[8]. The latter two are particularly useful for removing indirect co-evolutionary effects.

Given the high sensitivity of co-evolution results to the choice of methods, as recently shown[9], we focus mostly on the residue pairs that are confirmed by several methods, in particular DI and PSICOV.

**Perturbation Response Scanning:** PRS[10], the method that allows the calculation of the influence/sensitivity that each residue has on/to every other residue, was used to identify the most influential and most sensitive residues of DnaK. It is based on linear response theory[10,11]. The protein is modeled as an anisotropic network model (ANM)[12] where the nodes refer to individual residues (*N* of them), and springs to inter-residue interactions within a cutoff distance (12 Å). Node positions are identified with the coordinates of C^α^-atoms. The collective dynamics of the network is fully defined by the 3*N* × 3*N* Hessian matrix, **H**, whose elements (1 ≤ *i, j* ≤ N) are given by the second derivatives of the ANM potential[13]. The 3*N*-dimensional vector **ΔR** of node displacements in *response* to the application of a *perturbation* (a 3*N*-dimensional force vector **F)** obeys Hooke’s law $\mathbf{F}=\mathbf{H}\cdot\mathbf{\Delta R}$. As summarized in **Figure S1**, the idea in PRS is to exert a force of a given magnitude on the network, one residue at a time, and observe the response of the overall network. The force exerted on residue *i* is expressed as

| $\mathbf{F}^{(i)}=\left(\begin{matrix} 0 & 0 & 0 & \cdots& F_{x}^{(i)} & F_{x}^{(i)} & F_{x}^{(i)} & \cdots& 0 & 0 & 0 \end{matrix}\right)^{T}$, | (3) |
| --- | --- |

and the resulting response is

| $\mathbf{\Delta R}(i)=\mathbf{H}^{-\mathbf{1}}\mathbf{F}^{(i)}$ $,$ | (4) |
| --- | --- |

**ΔR**(*^i^*) is a *3N-*dimensional vector that describes the deformation of all the residues (in *N* blocks of dimension *3*, each) in response to $\mathbf{F}^{(i)}$. A metric for the response of residue *k* is the magnitude $\left\langle\left\|\mathbf{\Delta R}_{k}^{(i)}\right\|^{2}\right\rangle$ of the *k*^th^ block of **ΔR**(*^i^*) averaged over multiple **F**(*^i^*), expressed as the *ik*^th^ element of the *N*×*N* PRS matrix, **S**_PRS_. The elements of **S**_PRS_ refer to unit (or uniform) perturbing force. The response to unit deformation at each perturbation site is obtained by dividing each row by its diagonal value:

| $\overline{\mathbf{S}}_{\text{PRS}}=\left(\begin{matrix} 1/d_{1} & & 0 \\ & \ddots& \\ 0 & & 1/d_{N} \end{matrix}\right)\mathbf{S}_{\text{PRS}}$ | (5) |
| --- | --- |

Thus, the diagonal elements of $\bar{\text{S}}_{\text{PRS}}$ are all 1. The *i*^th^ row of $\bar{\text{S}}_{\text{PRS}}$ is referred to as the *influence profile* generated upon perturbing residue *i*; its average (over all receivers, *k*) is designated as $\left\langle\left(\Delta\text{R}^{\text{(}i\text{)}}\right)^{2}\right\rangle_{\inf}$. Similarly, the *k*^th^ column of $\bar{\text{S}}_{\text{PRS}}$ represents the *sensitivity profile* of residue *k*, in response to the perturbation of all residues; its average is denoted as $\left\langle\left(\Delta\text{R}_{k}\right)^{2}\right\rangle_{sen}$.

**Gaussian Network Model (GNM)** is used for evaluating the *mobility profile* *M_i_*(*^k^*) as a function of residue index *i*, for the normal mode *k,* following the protocol described in our previous work[14,15]. The homology model generated[16] for the ATP-bound conformer of DnaK, based on the structures resolved for an Hsp110 family member[17,18], is adopted to build conformer **D** (**Figure 1**). The network connectivity matrix **Γ** is evaluated using a cutoff distance of 7.3Å. The *k*^th^ eigenvector, **u(***^k^***)**, of **Γ** describes the *k*^th^ collective mode shape, and the corresponding eigenvalue, λ*_k_*, scales with the frequency of the mode and serves as a statistical weight. The low frequency modes (e.g. *k* ≤ 10), also called *soft modes*, usually dominate the *global dynamics* of the protein. The *i*^th^ element, [**u(***^k^***)**]*_i_*, of **u(***^k^***)** describes the displacement of residue *i* along the *k*^th^ mode; the plot of ([**u(***^k^***)**]_i_)^2^ as a function of residue index *i* defines the *mobility profile* *M_i_*(*^k^*) in mode *k*. The mobility profile averaged over a set of *m* modes is

| $\left.\left\langle M_{i}\right\rangle\right\vert_{m}=\frac{\sum_{k=1}^{m} \lambda_{k}^{-1}\left[u^{(k)}\right]_{i}^{2}}{\sum_{k=1}^{m} \lambda_{k}^{-1}}=\frac{\sum_{k=1}^{m} \lambda_{k}^{-1}M_{i}^{(k)}}{\sum_{k=1}^{m} \lambda_{k}^{-1}}$ | (6) |
| --- | --- |

Minima in the global modes usually refer to key mechanical sites (*global hinges*) that control the cooperative movements of subdomains, domains or subunits, whereas peaks indicate the most flexible or disordered regions, often involved in substrate recognition[19,20].

**Experimental methods**

#### Heat shock assay: The heat shock assay was performed as described previously[21]. Briefly, E. coli BB1553 cells (MC4100 ΔdnaK52::Cm^R^ sidB1, a gift from B. Bukau)[22] were transformed with either the empty pms119 vector or the pms119-dnaK vector containing the genes for the WT, L177A, or T417A variants. Overnight cultures (1 mL LB with 100 µg/mL ampicillin), which were inoculated from a single colony and incubated overnight at 30 °C, were diluted with LB to an optical density of 0.2 at 600 nm. The normalized growths were serially diluted five times (10-fold dilution factor) into sterile water pre-incubated at 43 °C, and plated onto LB plates containing 100 µg/mL ampicillin and pre-incubated at 43 °C. Plates were incubated at 43 °C for 16 hrs.

#### ΔSecB assay: The ΔSecB assay was performed as previously describe[23]. Briefly, *E. coli* GP502 (ΔsecB, P_BAD_-dnaKdnaJ)[24] cells were transformed with either the empty pms119 vector or the pms119-dnaK vector containing the genes for the WT, L177A, or T417A variants. Overnight cultures (1 mL LB with 50 µg/mL ampicillin, 50 µg/mL kanamycin, 0.5% w/v arabinose), which were inoculated from a single colony and incubated overnight at 30°C, were diluted with LB to an optical density at 600 nm of 0.4. The dilutions were used to inoculate 1 mL cultures in either 0.5% arabinose, 0.5% glucose, or 0.5% glucose plus 50 μm IPTG. Cultures were incubated at 30°C for 20 hrs at which time the optical density at 600 nm of each culture was measured.

#### Purification of proteins: WT, L177A and T417A DnaK variants were expressed from the pms119-dnaK vector in BB1553 cells and were purified as previously described[25] using a DEAE anion exchange column and an ATP-agarose affinity column. Pure DnaK was concentrated, buffer exchanged to remove unbound nucleotide, unfolded in 8M urea to remove remaining bound nucleotide, refolded into a 10-fold volumetric excess of 10 mM KPO_4_ 100 mM KCl 1mM EDTA pH 7.6 (PEK) buffer, buffer exchanged eight times into PEK to remove urea, and then buffer exchanged into 10 mM HEPES 100 mM KCl 5 mM MgCl_2_ pH 7.6 (HMK) buffer in a Centricon-30 concentrator (Amicon) prior to quick freezing with liquid nitrogen and storage at −80 °C. Protein concentrations were determined using an extinction coefficient of ε_280_=15.8 × 10^3^ M^−1^cm^−1^ [26] for all the DnaK variants.

#### Fluorescence assay: Spectra of the ATP-induced blue shift of W102 fluorescence were collected in 20 mM HEPES 100 mM KCl 5 mM MgCl_2_ pH 7.6 using an Photon Technology International Alpha Scan Fluorometer (Birmingham, NJ) as described previously[25]for 10 µM DnaK wild type, DnaK L177A, and DnaK T417A in the absence and presence of 1 mM ATP. Excitation wavelength was set to 295 nm and the emission spectra were recorded from 320 to 360 nm. The excitation and emission slits were set to 3nm and 1 nm respectively.

#### ATPase measurements: ATPase rates for DnaK wild type, DnaK L177A, and DnaK T417A were measured using the enzyme-coupled assay previously described[16,25] using a Biotek Synergy2 microplate reader. One or 10 µM DnaK was used for the peptide stimulated and basal rate measurements (respectively) in 40 mM HEPES 50 mM KCl 11 mM Mg(OAc)_2_) pH 7.6. Samples were incubated with 0.3 mM ATP at 30°C. The absorption at 340 nm, which corresponds to the oxidation of NADH and is coupled to the rate of ATP hydrolysis, is recorded for 40 min and the slope of the line is used to calculate the rate of ATP hydrolysis. The peptide stimulated rates were measured in the presence of 0.1 mM p5 or NR peptides. The pyruvate kinase and lactate dehydrogenase enzymes, phosophoenolpyruvate (PEP), and NADH were all purchased from Sigma Aldrich (St. Louis, MO)

**Supplemental Information on Results**

**Comparison of the global dynamics obtained for homology model and crystallographic structures resolved for ATP-bound DnaK.**

The structure of the ATP-bound, open SBD conformation of the Hsp70 cochaperone has been recently determined by Kityik *et al*.[27] and by Qi *et al*.[28]–PDB codes 4B9Q and 4JNE, respectively—by X-ray crystallography. In order to compare the computational results obtained with those structures and the homology model (HM) from earlier work, we compared (i) the GNM global modes produced by them, and (ii) the corresponding PRS maps. These comparisons are presented in **Figure S2**. Panel **A** shows the first two GNM modes of the three structures; we see that the mode shapes are almost indistinguishable i.e., the location of maxima and minima—a fundamental indicator of the sub-domains’ motion in GNM—is unchanged. Panel **B** shows the PRS maps obtained for the three structures; here again, a very similar behavior is observed, with differences due mostly to a scaling factor, while the profiles are closely maintained. Residues T417, D481 and G506 are observed to have minimal mobilities in both modes (see ordinate values close to zero), consistent with their participation in the global hinge center. They were indeed observed in 8 modes, out of 10 slowest (the cumulative effect of which is displayed in **Figure 2**), to have minimal displacements.

**Perturbation of the linker between the SBD and NBD of DnaK**

Results from perturbation-response scanning analysis performed for the linker residue V389 are shown in **Figure 4**. Panel **A** shows the most influential residues, and panel **B** displays their location on the structure, with V389 shown in *yellow* space-filling representation. They appear clustered in three groups**:** (i) residues on subdomain IA core (*red*), partially overlapping with the effectors detected in **Figure 3**, (ii) distal residues on the SBD β-sandwich (V381, I418, F426, A435, I462, I472 and K491, colored *purple*) again overlapping with the effectors detected in **Figure 3**, and (iii) a few residues (V205, V210, T215) on subdomain IIA (*green*), occupying a central location between the interface and ATP-binding site, in addition to some sequential and spatial neighbors to the linker in the NBD (*red* and *green*) and the SBD (L390 and A480, *purple*. The linker, thus, communicates with both domains, and even with distal regions in these domains. The distal residues in region (ii), A435, I462, I472, F426 and K491, may potentially serve as recognition/binding-site for the substrate.

**Sequence conservation and co-evolution analyses: Results**

**Figure S5** shows the heat maps for all the co-evolution methods used in this work, for the Hsp70 family; the same information is shown in **Figure 6A**, for the case of PSICOV. **Table S1** lists the amino acids that yield the top-ranking inter-domain values (measured by summing over each column/row of the co-evolution matrix), along with their sub-domain location and the methods that support these results. This is represented graphically in the **Figure 6B**.

**Perturbation-Response Scanning Results for L177**

Results from perturbation-response scanning analysis performed for the residue L177 are shown in **Figure S6.**

Reference List

1. Finn RD, Tate J, Mistry J, Coggill PC, Sammut SJ, *et al.* (2008) The Pfam protein families database. Nucleic Acids Res 36: D281-D288.

2. Bertelsen EB, Chang L, Gestwicki JE, Zuiderweg ER (2009) Solution conformation of wild-type E. coli Hsp70 (DnaK) chaperone complexed with ADP and substrate. Proc Natl Acad Sci U S A 106: 8471-8476.

3. Smith TF, Waterman MS (1981) Identification of common molecular subsequences. J Mol Biol 147: 195-197.

4. Dunn SD, Wahl LM, Gloor GB (2008) Mutual information without the influence of phylogeny or entropy dramatically improves residue contact prediction. Bioinformatics 24: 333-340.

5. Fodor AA, Aldrich RW (2004) Influence of conservation on calculations of amino acid covariance in multiple sequence alignments. Proteins 56: 211-221.

6. Weigt M, White RA, Szurmant H, Hoch JA, Hwa T (2009) Identification of direct residue contacts in protein-protein interaction by message passing. Proc Natl Acad Sci U S A 106: 67-72.

7. Morcos F, Pagnani A, Lunt B, Bertolino A, Marks DS, *et al.* (2011) Direct-coupling analysis of residue coevolution captures native contacts across many protein families. Proc Natl Acad Sci U S A 108: E1293-E1301.

8. Jones DT, Buchan DW, Cozzetto D, Pontil M (2012) PSICOV: precise structural contact prediction using sparse inverse covariance estimation on large multiple sequence alignments. Bioinformatics 28: 184-190.

9. Ackerman SH, Tillier ER, Gatti DL (2012) Accurate simulation and detection of coevolution signals in multiple sequence alignments. PLoS One 7: e47108.

10. Atilgan C, Atilgan AR (2009) Perturbation-response scanning reveals ligand entry-exit mechanisms of ferric binding protein. PLoS Comput Biol 5: e1000544.

11. Ikeguchi M, Ueno J, Sato M, Kidera A (2005) Protein structural change upon ligand binding: linear response theory. Phys Rev Lett 94: 078102.

12. Eyal E, Yang LW, Bahar I (2006) Anisotropic network model: systematic evaluation and a new web interface. Bioinformatics 22: 2619-2627.

13. Atilgan AR, Durell SR, Jernigan RL, Demirel MC, Keskin O, *et al.* (2001) Anisotropy of fluctuation dynamics of proteins with an elastic network model. Biophys J 80: 505-515.

14. Yang LW, Rader AJ, Liu X, Jursa CJ, Chen SC, *et al.* (2006) oGNM: online computation of structural dynamics using the Gaussian Network Model. Nucleic Acids Res 34: W24-W31.

15. Bahar I, Atilgan AR, Erman B (1997) Direct evaluation of thermal fluctuations in proteins using a single-parameter harmonic potential. Fold Des 2: 173-181.

16. Smock RG, Rivoire O, Russ WP, Swain JF, Leibler S, *et al.* (2010) An interdomain sector mediating allostery in Hsp70 molecular chaperones. Mol Syst Biol 6: 414.

17. Liu Q, Hendrickson WA (2007) Insights into Hsp70 chaperone activity from a crystal structure of the yeast Hsp110 Sse1. Cell 131: 106-120.

18. Schuermann JP, Jiang J, Cuellar J, Llorca O, Wang L, *et al.* (2008) Structure of the Hsp110:Hsc70 nucleotide exchange machine. Mol Cell 31: 232-243.

19. Bahar I, Lezon TR, Yang LW, Eyal E (2010) Global dynamics of proteins: bridging between structure and function. Annu Rev Biophys 39: 23-42.

20. Liu Y, Gierasch LM, Bahar I (2010) Role of Hsp70 ATPase domain intrinsic dynamics and sequence evolution in enabling its functional interactions with NEFs. PLoS Comput Biol 6.

21. Clerico EM, Zhuravleva A, Smock RG, Gierasch LM (2010) Segmental isotopic labeling of the Hsp70 molecular chaperone DnaK using expressed protein ligation. Biopolymers 94: 742-752.

22. Bukau B, Walker GC (1990) Mutations altering heat shock specific subunit of RNA polymerase suppress major cellular defects of E. coli mutants lacking the DnaK chaperone. EMBO J 9: 4027-4036.

23. Smock RG, Blackburn ME, Gierasch LM (2011) Conserved, disordered C terminus of DnaK enhances cellular survival upon stress and DnaK in vitro chaperone activity. J Biol Chem 286: 31821-31829.

24. Ullers RS, Ang D, Schwager F, Georgopoulos C, Genevaux P (2007) Trigger Factor can antagonize both SecB and DnaK/DnaJ chaperone functions in Escherichia coli. Proc Natl Acad Sci U S A 104: 3101-3106.

25. Montgomery DL, Morimoto RI, Gierasch LM (1999) Mutations in the substrate binding domain of the Escherichia coli 70 kDa molecular chaperone, DnaK, which alter substrate affinity or interdomain coupling. J Mol Biol 286: 915-932.

26. Jordan R, McMacken R (1995) Modulation of the ATPase activity of the molecular chaperone DnaK by peptides and the DnaJ and GrpE heat shock proteins. J Biol Chem 270: 4563-4569.

27. Kityk R, Kopp J, Sinning I, Mayer MP (2012) Structure and dynamics of the ATP-bound open conformation of Hsp70 chaperones. Mol Cell 48: 863-874.

28. Qi R, Sarbeng EB, Liu Q, Le KQ, Xu X, *et al.* (2013) Allosteric opening of the polypeptide-binding site when an Hsp70 binds ATP. Nat Struct Mol Biol 20: 900-907.
